# Compound *Abrus cantoniensis* Extract Alleviates AFB_1_‐Induced Liver Injury by Regulating PI3K‐AKT Pathway and Gut Microbiota/Butyrate Metabolism

**DOI:** 10.1002/advs.77889

**Published:** 2026-09-27

**Authors:** Hongjie Hu, Ming cheng, Shiqi Zheng, Huixin Liu, Xiaofang Wei, Wenwen Yang, Hongchun Yang, Liuyang Yuan, Li Jiang, Jie Chen, Linglong Zhao, Yanchao Guo, Hongbin Si

**Affiliations:** ^1^ College of Animal Science and Technology Guangxi Key Laboratory of Animal Breeding Disease Control and Prevention Guangxi Grass Station Guangxi University Nanning Guangxi China; ^2^ School of Liberal Arts Guangxi Minzu University Nanning China

**Keywords:** AFB_1_‐induced liver injury, compound *Abrus cantoniensis*, gut‐butyrate‐liver axis, main components, PI3K‐AKT pathway

## Abstract

Aflatoxin B_1_ (AFB_1_) is a potent hepatotoxin commonly ingested through contaminated food. *Abrus cantoniensis* Hance, a traditional Chinese medicinal herb, is commonly included in hepatoprotective compound prescriptions, yet its protective role against AFB_1_‐induced hepatic injury remains to be elucidated. Here, network pharmacology combined with molecular docking suggested that the compound *Abrus cantoniensis* extract (ACCE) attenuates AFB_1_‐induced hepatotoxicity via modulation of the PI3K‐AKT signaling pathway. This was validated through integrated transcriptomic, microbiomic, metabolomic, and in vivo/in vitro analyses. In vivo, ACCE ameliorated hepatic and intestinal damage in AFB_1_‐exposed mice, modulated PI3K‐AKT and TLR4/NF‐κB pathways, reshaped gut microbiota by enriching *Lachnospiraceae_NK4A136_group*, and increased short‐chain fatty acids (SCFAs), particularly butyrate. In vitro, ACCE activated PI3K‐AKT and suppressed TLR4/NF‐κB signaling in HepG2 cells, effects diminished by pathway inhibitors. Butyrate alone also protected against AFB_1_‐induced injury via TLR4/NF‐κB inhibition, an effect similarly reversed by inhibitors. Notably, combined treatment with ACCE or its main components and butyrate yielded superior protection. Collectively, these findings suggest that ACCE can alleviate AFB_1_‐induced liver injury by regulating the PI3K‐AKT pathway and the gut microbiota and its metabolite butyrate.

## Graphical abstract

ACCE has a good alleviating effect on AFB_1_‐induced liver injury, and this alleviating effect is related to the PI3K‐AKT pathway regulated by ACCE and the butyrate metabolism of gut microbiota.

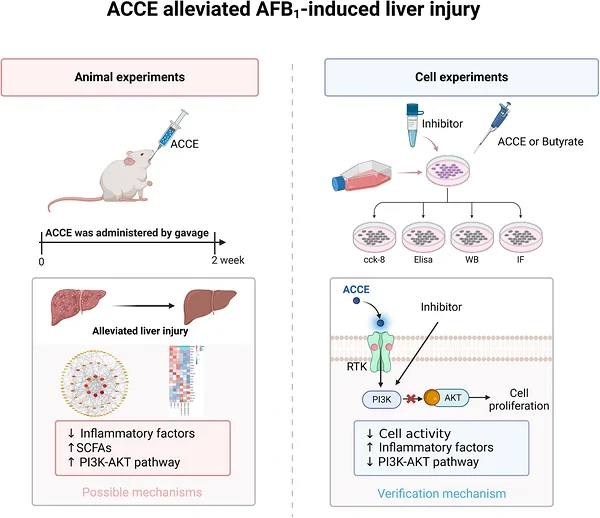

## 1 Introduction

Aflatoxin B_1_ (AFB_1_), one of the most potent naturally occurring mycotoxins, widely contaminates food and animal feed globally. It poses severe threats to global food security and public health, causing considerable financial losses to the animal husbandry industry [1]. The liver bears the brunt of AFB_1_ toxicity. After metabolic activation by cytochrome P450 enzymes, AFB_1_ generates highly electrophilic epoxides that bind covalently to nucleic acids and proteins, inducing hepatocyte injury and genetic mutations [2]. Epidemiological studies have confirmed that AFB_1_ exposure is a class I carcinogen associated with hepatocellular carcinoma (HCC). Liver injury resulting from AFB_1_ contamination has become a significant health burden, particularly in developing countries [3].

The concept of the gut‐liver axis describes a two‐way signaling system between the intestine and the liver, which is critical for maintaining hepatic metabolism and intestinal homeostasis [4]. Gut microbial metabolites, including short‐chain fatty acids (SCFAs), are carried through the portal vein to the liver, where they are involved in modulating host metabolism and immune responses. An intact intestinal mucosa is regarded as the primary barrier that impedes harmful agents from accessing the liver [5, 6]. However, exposure to AFB_1_ can severely disrupt the homeostasis of the gut‐liver axis. AFB_1_ has been reported to suppress the expression of intestinal tight junction proteins (e.g., ZO‐1, Occludin, and Claudin‐1), thereby weakening the gut barrier, raising mucosal permeability, and inducing dysbiosis of the gut microbiota [7, 8, 9]. These changes facilitate the translocation of harmful substances, such as lipopolysaccharides (LPS), into the portal circulation, subsequently activating inflammatory pathways in the liver (e.g., TLR4/NF‐κB signaling pathway), exacerbating liver injury. Therefore, the disruption of the gut‐liver axis is considered one of the key pathways for AFB_1_‐induced hepatotoxicity. The gut microbiota, as a significant player in the gut‐liver axis, produces SCFAs, particularly butyrate, which can exert anti‐inflammatory actions through suppression of the TLR4/NF‐κB pathway and maintaining intestinal barrier function [10].

Previous studies have reported that the dysregulation of multiple signaling pathways or targets is involved in AFB_1_‐induced liver injury, including the PI3K‐AKT, PXR, NRF2, and NLRP3 pathways [11, 12, 13]. Among these, the PI3K‐AKT pathway is a core regulator of cell survival, proliferation, and apoptosis. Activation of the PI3K‐AKT pathway promotes hepatocyte survival and counteracts pro‐apoptotic signals, whereas AFB_1_ exposure suppresses this signaling cascade, thereby enhancing hepatocyte apoptosis and aggravating hepatic injury [14]. Meanwhile, the TLR4/NF‐κB pathway is a central mediator of inflammatory responses. When the intestinal barrier is compromised, LPS gains access to the portal vein and stimulates hepatic TLR4, thereby initiating NF‐κB activation and driving the expression of pro‐inflammatory cytokines including TNF‐α, IL‐1β, and IL‐6 [15]. Therefore, hepatoprotective strategies that can simultaneously regulate the PI3K‐AKT and TLR4/NF‐κB pathways may have potential for alleviating AFB_1_‐induced liver injury.

Originating from the Lingnan area of China, *Abrus cantoniensis* Hance is a geo‐authentic medicinal herb that contains high levels of flavonoids, saponins, and polysaccharides. These bioactive compounds confer multiple therapeutic properties, such as anti‐inflammatory, antioxidant, and immunomodulatory activities [16]. In traditional Chinese medicine (TCM) practice, *Abrus cantoniensis* is incorporated into over 100 compound formulations due to its capacity to soothe the liver, promote bile secretion, detoxify, and resolve stasis, with its hepatoprotective efficacy widely acknowledged. However, whether the compound *Abrus cantoniensis* confers protection against AFB_1_‐induced hepatic injury remains unknown, and the associated mechanisms likewise require further investigation.

Therefore, the current investigation was designed to comprehensively explore the hepatoprotective efficacy and mechanistic basis of the compound *Abrus cantoniensis* extract (ACCE) against AFB_1_‐related hepatic injury. To achieve this goal, we employed a combination of various research methods, including network pharmacology, molecular docking, transcriptomics, microbiomics, and targeted metabolomics, alongside in vivo and in vitro research systems [17]. We strive to reveal the mechanisms by which ACCE alleviates AFB_1_‐induced liver injury from multiple angles and levels, providing substantial experimental evidence for its development as a hepatoprotective drug.

## 2 Results

### 2.1 ACCE Effectively Alleviates AFB_1_‐Induced Liver Injury

**FIGURE 1:**
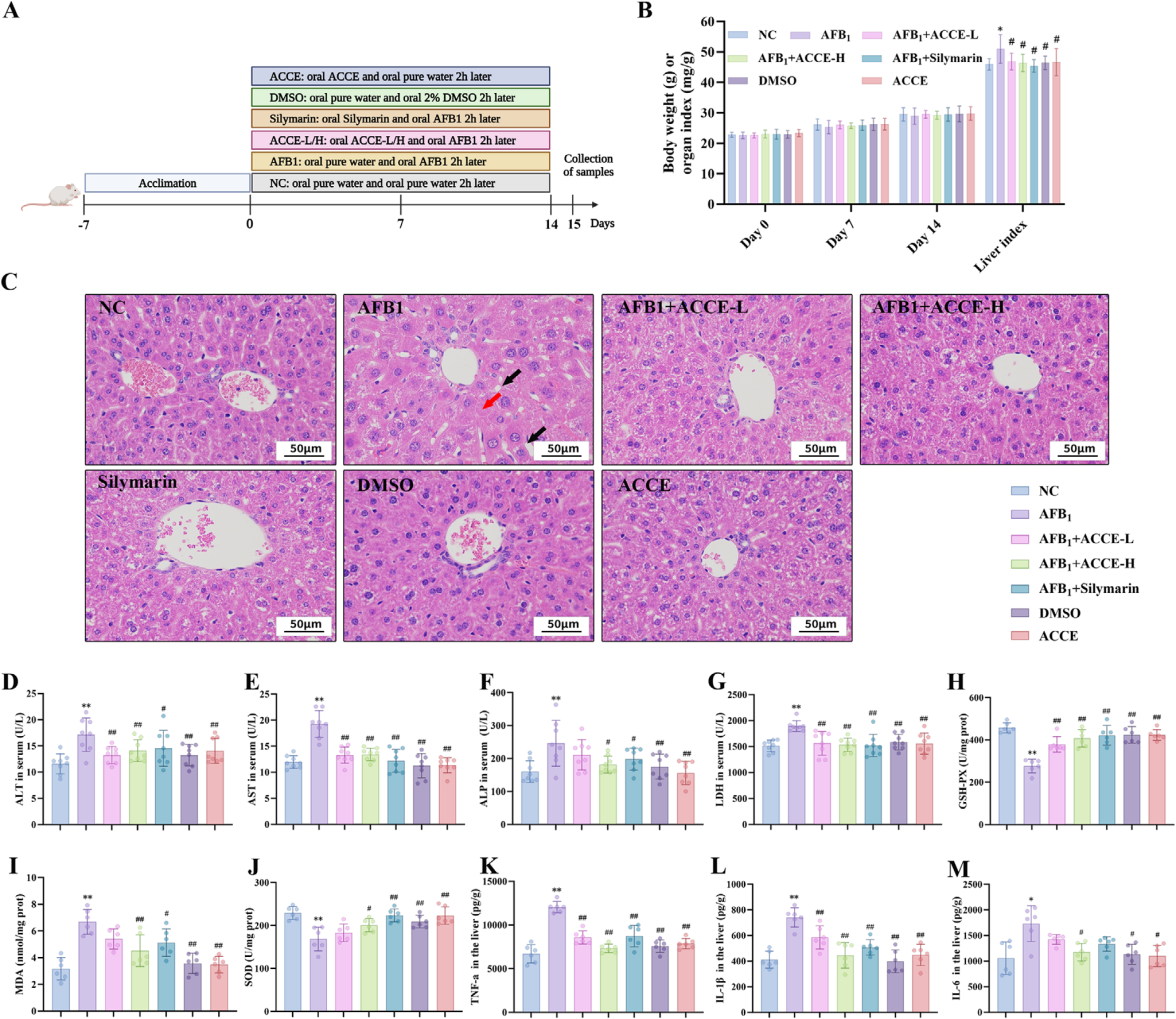
Effects of AFB_1_ exposure and ACCE intervention on growth status, liver structure and function, oxidation, and inflammation in mice. (A) Schematic of the treatment of AFB_1_ and ACCE in animal experiments. (B) Body weight and liver organ index, *n* = 11. (C) HE staining of the liver. (400×). (D–G) Detection results of liver function index, *n* = 8. (H–J) Related indicators of oxidative injury, *n* = 6. (K–M) Related indicators of liver inflammation, *n* = 6. Statistical analysis was performed by One‐Way ANOVA with Tukey post‐test (B, D–L) or Welch's ANOVA with Games‐Howell post‐test (B, M). ^*^ *p* < 0.05, ^**^ *p* < 0.01, versus NC; ^#^ *p* < 0.05, ^##^ *p* < 0.01, versus AFB_1_.

To clarify the effect of ACCE treatment on AFB_1_‐induced liver injury, mouse body weights and liver indices in each group were recorded (Figure 1B). Prior to AFB_1_ exposure, body weights did not differ significantly among groups. After 7 and 14 days of exposure, mice in the AFB_1_ group showed a slower increase in body weight compared to other groups, although these differences were not statistically significant. The liver index in the AFB_1_ group was significantly higher than in the NC group, whereas intervention with ACCE and silymarin effectively reversed this increase. H&E staining revealed that liver tissues from the NC group exhibited normal hepatic structures. Conversely, the AFB_1_ group displayed disrupted hepatic plate arrangement, hepatocytes of varying sizes, ballooning degeneration with vacuoles of varying dimensions (black arrows), as well as hepatocyte necrosis and nuclear fragmentation or dissolution (red arrows) (Figure 1C). In comparison, liver tissues from ACCE‐L, ACCE‐H, and silymarin groups showed partial cellular disorganization, significantly attenuated vacuolar degeneration, and improved nuclear morphology. The DMSO control and ACCE‐alone groups exhibited intact hepatic cord arrangements and normal cell morphology, without significant differences from the NC group, indicating that neither 2% (v/v) DMSO nor ACCE alone induced liver injury.

Serum activities of liver function markers (ALT, AST, ALP, LDH) were significantly elevated after AFB_1_ exposure compared to the NC group, indicating liver injury. Treatment with ACCE and silymarin significantly reduced these enzyme activities, with the most notable improvement observed in the ACCE‐H group (Figure 1D–G). Regarding oxidative stress, hepatic MDA levels were significantly increased, while activities of SOD and GSH‐Px were significantly reduced in the AFB_1_ group. Treatment with ACCE‐L, ACCE‐H, and silymarin reversed these changes to varying degrees (Figure 1H–J). In addition, the elevated levels of inflammatory factors TNF‐α, IL‐1β, and IL‐6 in liver tissues induced by AFB1 exposure were significantly alleviated by treatment with ACCE‐L, ACCE‐H, and Silymarin. Collectively, these findings indicated that AFB_1_ exposure caused oxidative and inflammatory liver injury, which was effectively alleviated by ACCE intervention.

### 2.2 ACCE Ameliorates AFB_1_‐Induced Intestinal Injury and Systemic Inflammation

**FIGURE 2:**
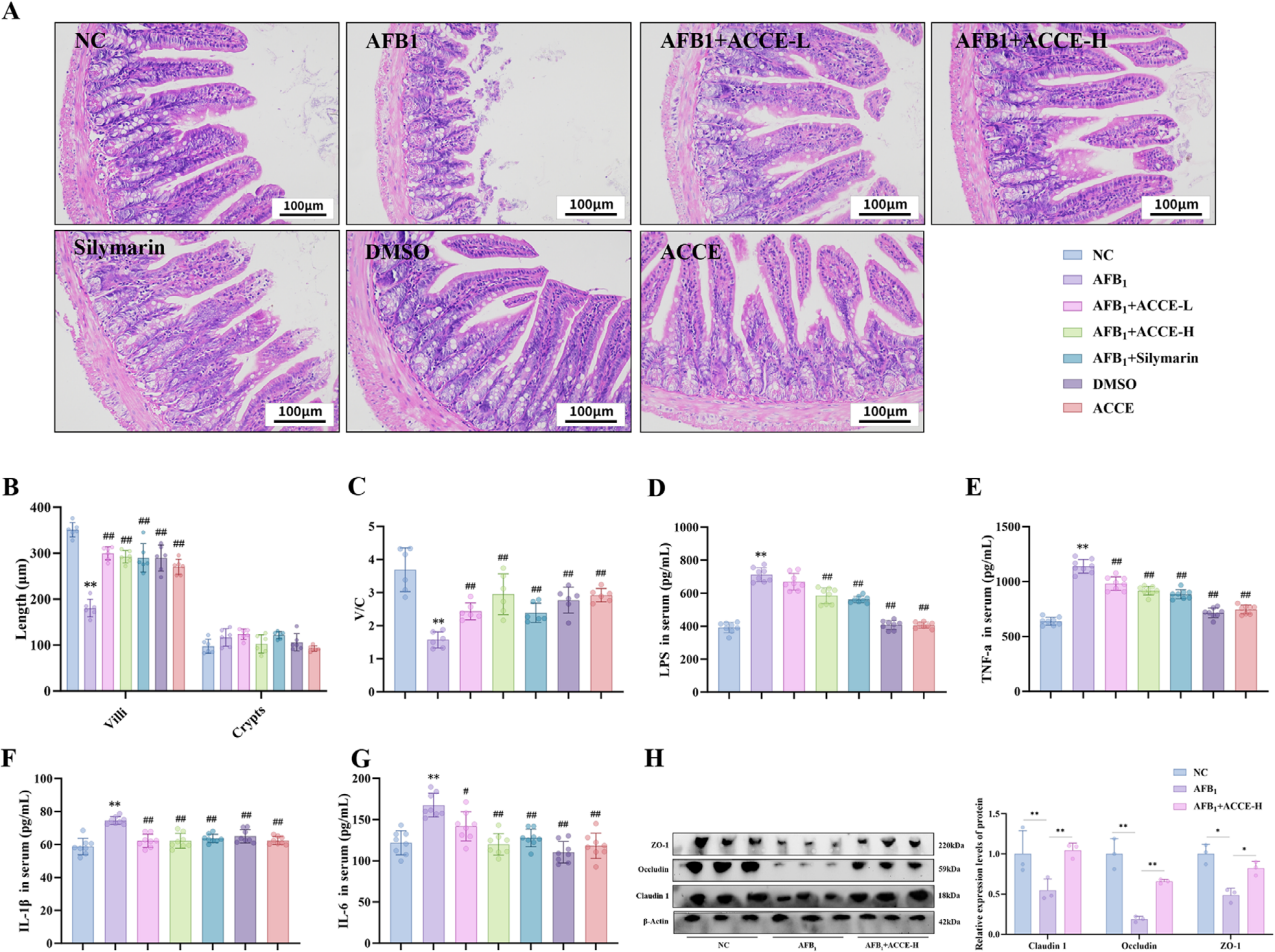
The effects of AFB_1_ exposure and ACCE intervention on the intestinal structure, intestinal barrier, and serum inflammation in mice. (A) HE staining of the ileum (200×). (B) The length of the villi and crypts in the ileum, *n* = 6. (C) The ratio of villi to crypts in the ileum (V/C), *n* = 6. (D) The level of LPS in the serum, *n* = 8. (E–G) Inflammation‐related indicators in serum, *n* = 8. (H) Tight junction protein expression levels in the ileum, *n* = 3. Statistical analysis was performed by One‐Way ANOVA with Tukey post‐test (B, E–H) or Welch's ANOVA with Games‐Howell post‐test (B–D). ^*^ *p* < 0.05, ^**^ *p* < 0.01, versus NC; ^#^ *p* < 0.05, ^##^ *p* < 0.01, versus AFB_1_.

To investigate the effects of ACCE treatment on intestinal injury induced by AFB_1_, ileal tissues were examined by H&E staining (Figure 2A). Compared with the NC group, mice in the AFB_1_ group showed significantly decreased villus height and villus‐to‐crypt (V/C) ratio, indicating impaired intestinal absorption. These pathological changes were significantly mitigated in the ACCE‐L, ACCE‐H, and silymarin groups, with the V/C ratio substantially restored (Figure 2B,C). In addition, serum levels of LPS were markedly increased following AFB_1_ exposure, while ACCE and silymarin significantly reduced these levels. Serum inflammatory cytokines, indicative of systemic inflammation, demonstrated consistent changes (Figure 2D–G). The expression levels of tight junction proteins (ZO‐1, Occludin, Claudin 1), which are indicative of intestinal mucosal integrity, were found to be down‐regulated by AFB_1_. Notably, this down‐regulation was significantly reversed following treatment with ACCE‐H (Figure 2H). Collectively, these results indicated that AFB_1_ exposure caused intestinal injury, endotoxin translocation into the bloodstream, and systemic inflammation, whereas ACCE intervention effectively attenuated these pathological alterations.

### 2.3 Network Pharmacology Combined with Transcriptomics Analysis of the Potential Mechanism by which ACCE Alleviates Liver Injury

The composition of ACCE was analyzed using UPLC‐MS/MS (Figure S1A,B). Total ion chromatograms (TICs) of pooled quality control (QC) samples in negative and positive ion modes are presented in Figure S1C,D, respectively (Figure S1C,D). A total of 24 main components were identified in ACCE, including salvianolic acid B, neochlorogenic acid, chlorogenic acid, isoschaftoside, and butyl isobutyl phthalate, among others. Detailed information is provided in the supplementary table (Table S1).

**FIGURE 3:**
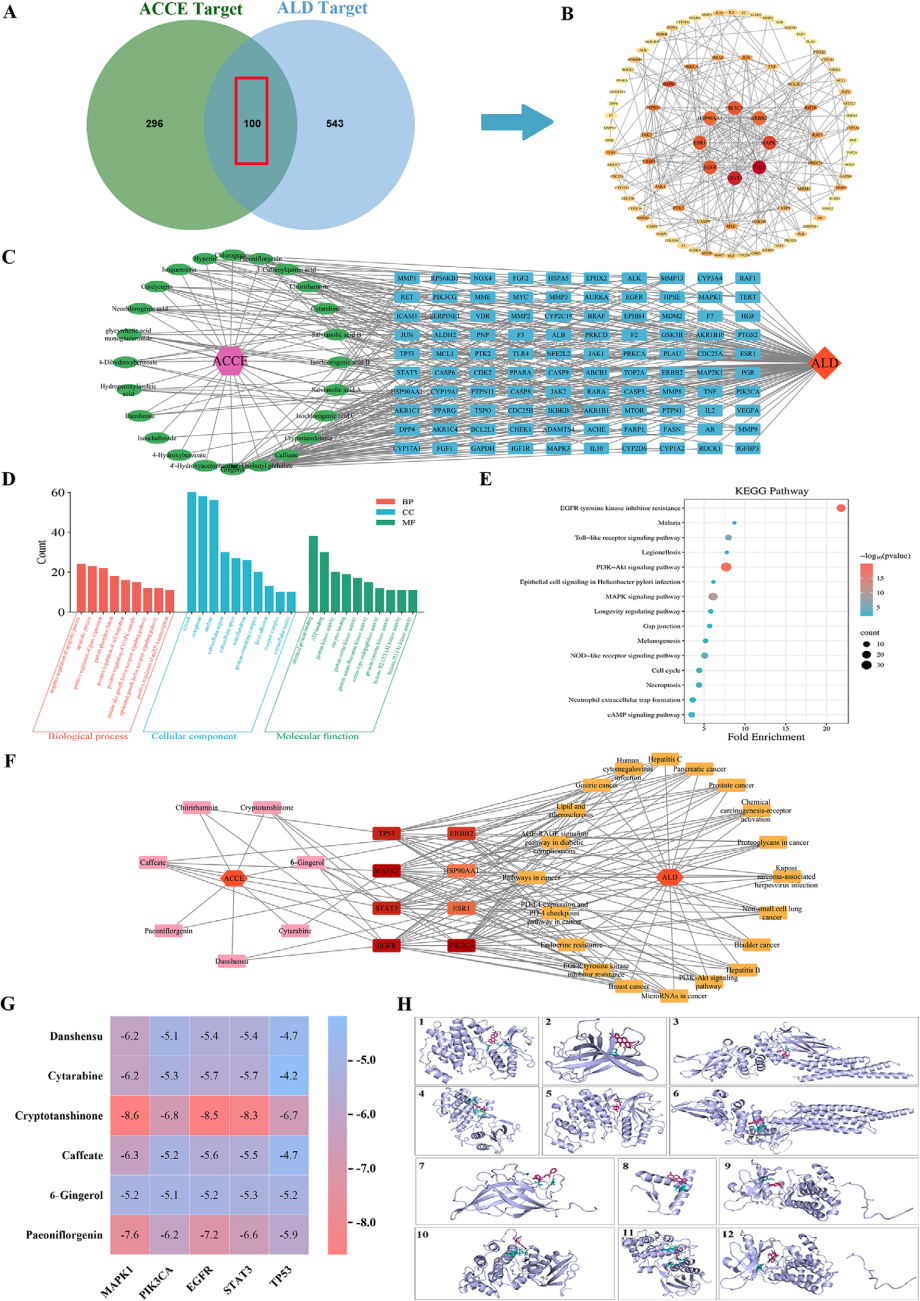
Results of network pharmacology and molecular docking analysis. (A) Venn diagram of target genes of active ingredients and disease‐related genes. (B) Protein‐protein interaction network (PPI). (C) The “drug‐active ingredient‐potential target” network. (D) GO enrichment analysis of 100 potential targets. (E) KEGG enrichment analysis of 100 potential targets. (F) The “drug‐component‐target‐pathway‐disease” network was constructed from core targets and their corresponding components and pathways. (G) Heat map of molecular docking results. (H) Molecular docking map of the top 12 binding energies of the target protein and the active ingredient.

Based on the 24 primary ACCE components identified by UPLC‐MS/MS, target prediction using the SwissTargetPrediction database yielded 396 non‐redundant targets after deduplication. A total of 643 targets associated with AFB_1_‐induced liver disease (ALD) were retrieved from the GeneCards and OMIM databases. The intersection of component targets and disease‐related targets yielded 100 potential therapeutic targets (Figure 3A). A “drug‐active component‐potential target” network was constructed using these 100 targets and their corresponding 24 active components, and visualized with Cytoscape 3.10.2 (Figure 3C). Among the components, [6]‐gingerol, caffeate, cryptotanshinone, isochlorogenic acid C, salvianolic acid B, and cytarabine ranked highest based on degree values, indicating their potential as key active constituents of ACCE against AFB_1_‐induced liver injury. The 100 potential targets were further imported into the STRING database to generate a protein‐protein interaction (PPI) network, visualized using Cytoscape 3.10.2 (Figure 3B). The top eight targets (including TP53, STAT3, MAPK1, and EGFR) occupied central positions in the network, suggesting their critical roles in the regulation of AFB_1_‐induced liver injury. Additionally, these 100 potential targets underwent GO and KEGG enrichment analyses using the DAVID database (Figure 3D,E). GO analysis indicated that ACCE might alleviate AFB_1_‐induced liver injury through biological processes such as PI3K‐AKT signaling, MAPK cascade, gene expression regulation, and apoptosis modulation. KEGG pathway enrichment further suggested the involvement of multiple pathways, particularly the PI3K‐AKT and MAPK signaling pathways.

A drug‐component‐target‐pathway‐disease network was constructed based on the core targets, their corresponding active components, and relevant pathways (Figure 3F). Among these, five genes (MAPK1, PIK3CA, EGFR, STAT3, and TP53) rank high in Degree values, suggesting that they may serve as key targets. Molecular docking was then performed on the five core targets and their corresponding active components to facilitate virtual screening and predict potential binding modes. The heatmap results revealed that the binding energies between major active components and core targets were all below −4.0 kcal/mol. This value is a commonly used threshold in virtual screening; values below this threshold suggest that there may be good binding activity between the two. This indicates that these components may play a crucial role in alleviating AFB_1_‐induced liver injury (Figure 3G). The 12 top‐ranked target–component combinations, based on binding energies, were selected for 3D structural visualization (Figure 3H). Of course, both molecular docking and network pharmacology analyses are prediction results and do not constitute direct evidence of target‐component binding.

**FIGURE 4:**
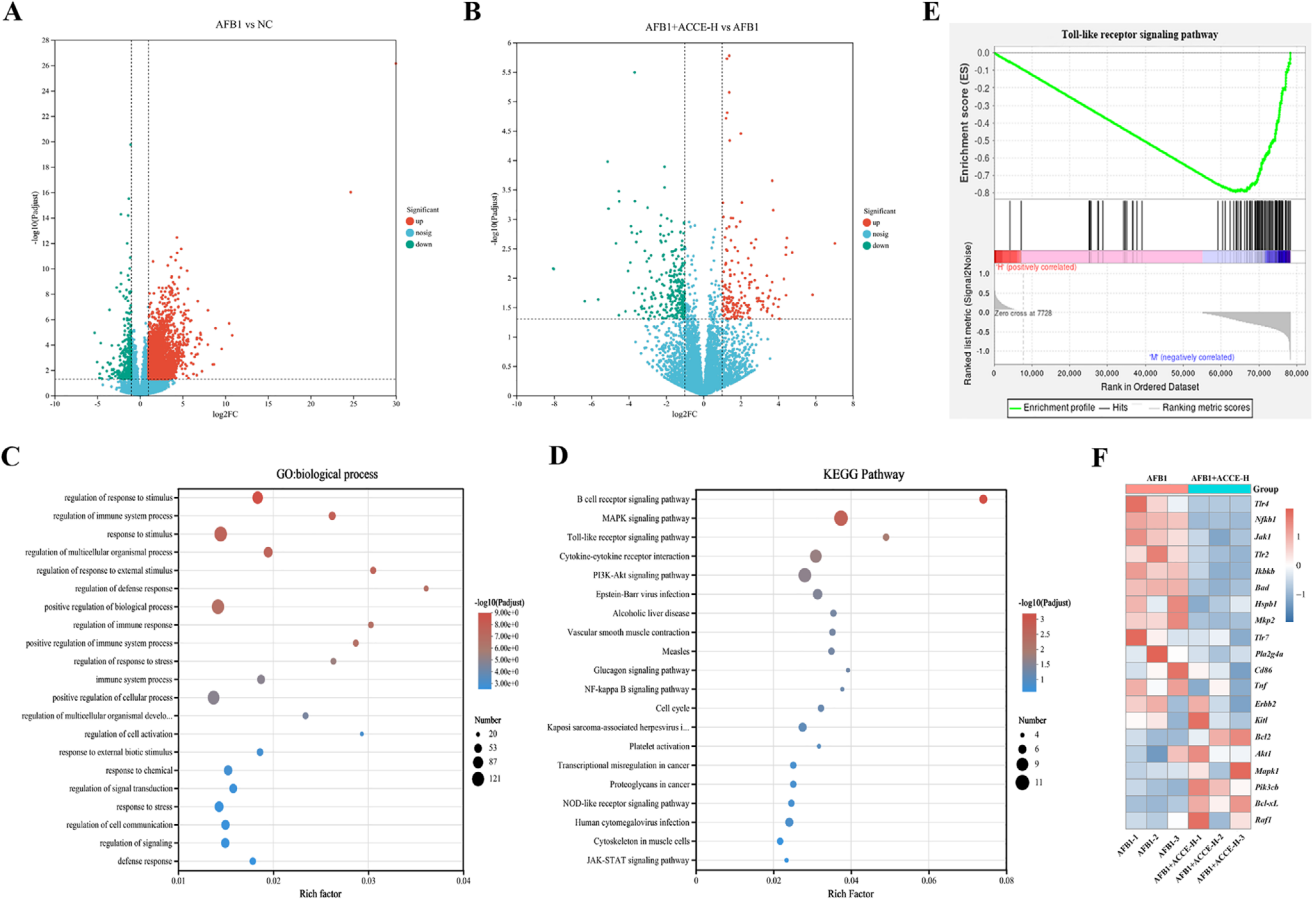
RNA‐seq results of mouse liver. (A, B) Volcano plot of differentially expressed genes (DEGs). (C, D) GO enrichment analysis and KEGG enrichment analysis of DEGs. (E) Significantly enriched signaling pathways were analyzed by GSEA. (F) Clustering heatmap of related genes involved in signaling pathway regulation.

To further elucidate the mechanisms underlying the hepatoprotective effects of ACCE against AFB_1_‐induced injury, mouse liver gene expression profiles were analyzed using RNA‐seq. DEGs between the AFB_1_ and NC groups, as well as between the ACCE‐H and AFB_1_ groups, were visualized as volcano plots (Figure 4A,B). GO and KEGG enrichment analyses demonstrated that DEGs were primarily enriched in biological processes such as regulation of stress responses, positive regulation of cellular processes, and regulation of inflammatory responses (Figure 4C). Pathways including *Pi3k‐Akt*, *Mapk*, and Toll‐like receptor (TLR) signaling were significantly enriched (Figure 4D). Gene Set Enrichment Analysis (GSEA) further confirmed significant enrichment of the TLR signaling pathway (Figure 4E). Additionally, a heatmap of related genes involved in these three pathways was constructed (Figure 4F).

### 2.4 ACCE Restores AFB1‐Induced Gut Microbiota Dysbiosis

**FIGURE 5:**
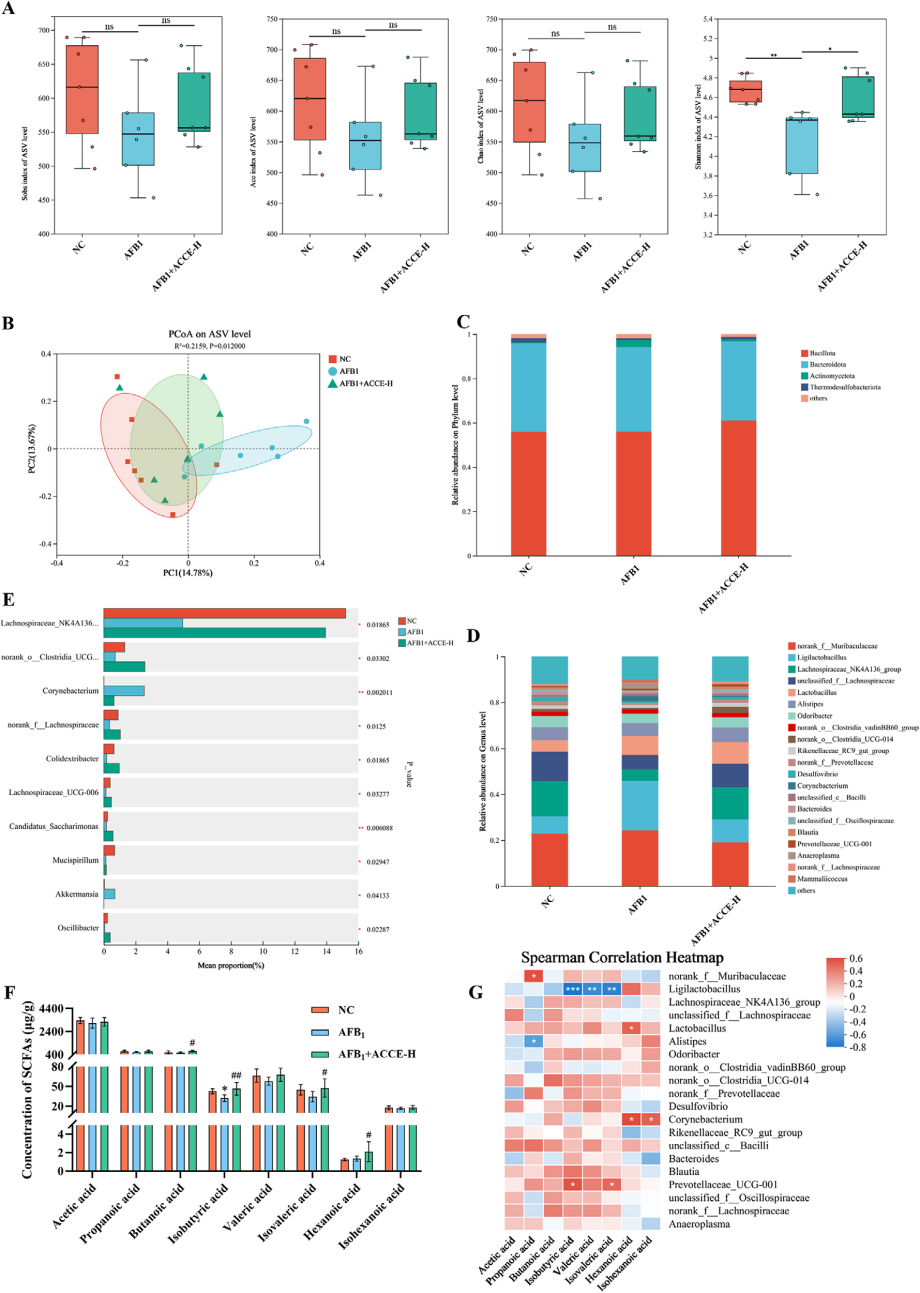
Results of 16S rRNA sequencing and SCFAs assay of mice intestinal contents. (A) Analysis of α‐diversity of gut microbiota. (B) Analysis of β‐diversity of gut microbiota. (C–E) Analysis of gut microbiota at the phylum and genus levels. (F) The concentration of SCFAs, *n* = 6. (G) Correlation analysis between SCFAs levels and gut microbial abundance. Statistical analysis was performed by One‐Way ANOVA with Tukey post‐test (F). ^*^ *p* < 0.05, ^**^ *p* < 0.01, versus NC; ^#^ *p* < 0.05, ^##^ *p* < 0.01, versus AFB_1_; ns, no significance.

To explore the effects of ACCE treatment on gut microbiota and the intestinal mucosal barrier, 16S rRNA gene sequencing was performed on mouse intestinal content samples. α‐Diversity analysis revealed decreased values of Sobs, Ace, Chao, and Shannon indices in the AFB_1_ group, whereas these parameters were reversed by ACCE‐H treatment, with the Shannon index significantly increased (Figure 5A). β‐Diversity analysis indicated that ACCE‐H treatment corrected the disrupted gut microbiota structure induced by AFB_1_, making it closer to that observed in the NC group (Figure 5B). At the phylum level, the relative abundance of Actinomycetota increased following AFB_1_ exposure (Figure 5C). At the genus level, significant alterations were observed in the abundances of SCFA‐producing bacteria (e.g., *Lachnospiraceae_NK4A136_group*, *norank_o_Clostridia_UCG‐014*, *norank_f_Lachnospiraceae*) and potentially pathogenic bacteria (e.g., *Corynebacterium*) in the AFB_1_ group, whereas ACCE‐H treatment effectively restored their abundances (Figure 5D,E). Correspondingly, levels of short‐chain fatty acids (SCFAs), including acetate, propionate, and butyrate, were normalized in the ACCE‐H group, with butyrate concentrations positively correlating with abundances of *Lachnospiraceae_NK4A136_group* (Figure 5F,G). Similarly, peripheral blood butyrate levels were significantly higher in the ACCE‐H group than in the AFB1 group (Figure S4).

### 2.5 Potential Mechanism of ACCE in Alleviating Liver Injury

**FIGURE 6:**
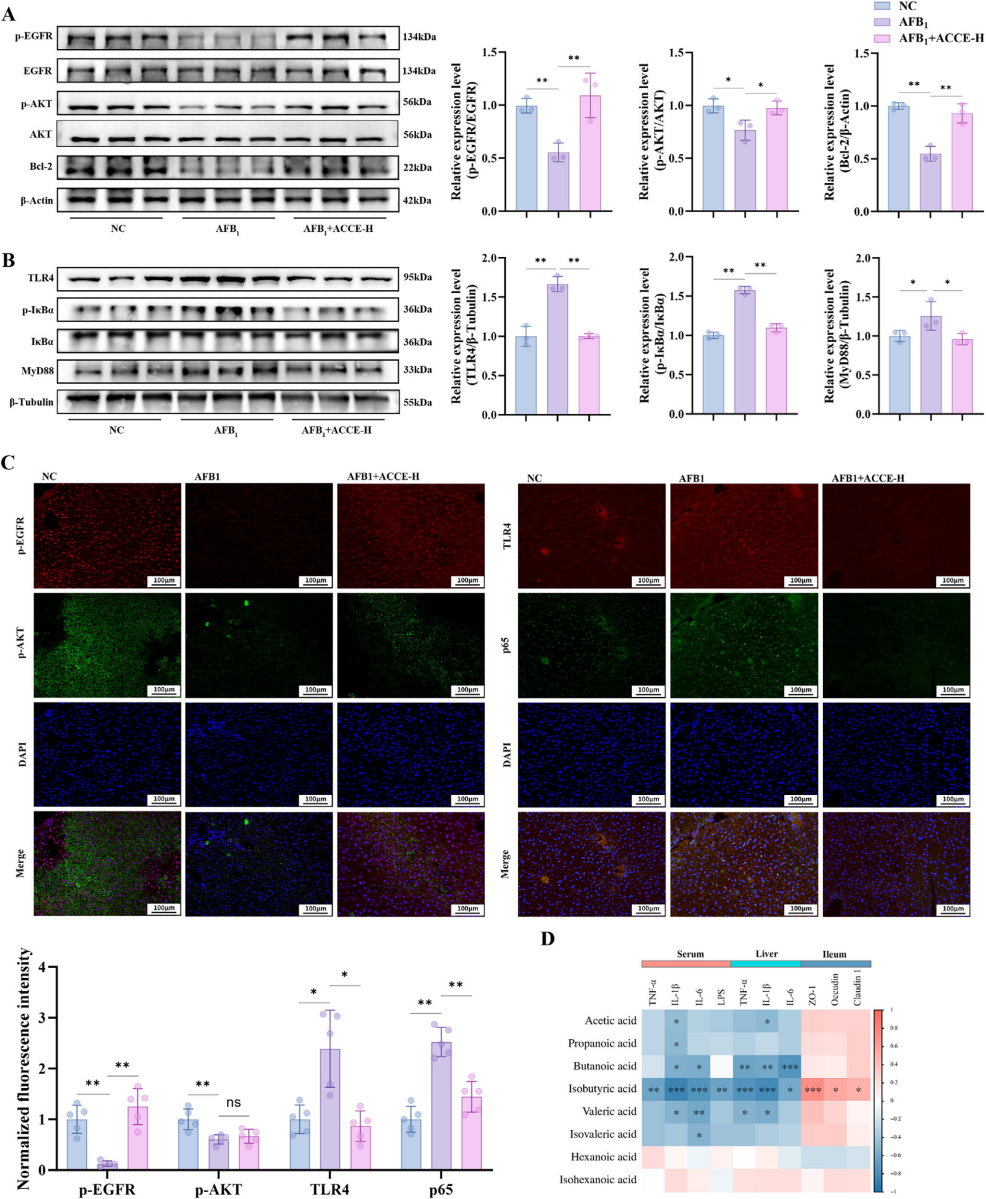
The expression status of key proteins in the PI3K‐AKT pathway and the TLR4/NF‐κB pathway. (A) Western blotting detection of key proteins in the PI3K‐AKT pathway, *n* = 3. (B) Western blotting detection of key proteins in the TLR4/NF‐κB pathway, *n* = 3. (C) Results of immunofluorescence detection of key proteins (400×). (D) Correlation plots between SCFAs levels and various indicators in serum, liver, and ileum. Statistical analysis was performed by One‐Way ANOVA with Tukey post‐test (A–C) or Welch's ANOVA with Games‐Howell post‐test (C). ^*^ *p* < 0.05; ^**^ *p* < 0.01; ns, no significance.

Based on results from PPI network analysis, molecular docking, KEGG pathway enrichment, and transcriptome sequencing, it was hypothesized that the PI3K‐AKT pathway (associated with cell proliferation) and the TLR4/NF‐κB pathway (associated with inflammation) are critically involved in ACCE‐mediated alleviation of AFB_1_‐induced liver injury. Western blot analysis demonstrated that ACCE‐H treatment significantly increased protein expression levels of phosphorylated EGFR (p‐EGFR), phosphorylated AKT (p‐AKT), and Bcl‐2 within the PI3K‐AKT pathway, while significantly decreasing the expression of TLR4, MyD88, and phosphorylated IκBα (p‐IκBα) within the TLR4/NF‐κB pathway (Figure 6A,B). Immunofluorescence staining of liver sections confirmed these findings, indicating that ACCE‐H treatment activated p‐EGFR and p‐AKT and inhibited the expression of TLR4 and NF‐κB p65 (Figure 6C).

Furthermore, correlation analysis revealed that SCFA levels, particularly butyrate, were significantly negatively correlated with inflammatory factors (e.g., TNF‐α) and serum LPS levels, while positively correlated with the expression levels of intestinal tight‐junction proteins (ZO‐1, Occludin, Claudin 1). Collectively, these data support the hypothesis that the gut‐butyrate‐liver axis may play a pivotal role in ACCE‐mediated alleviation of AFB_1_‐induced liver injury (Figure 6D).

### 2.6 Validation via Cell Experiments

Integrating preliminary experimental findings and existing literature, the following hypothesis was proposed: ACCE acts through two complementary mechanisms. On one hand, its active components directly target hepatocytes, activating the PI3K‐AKT survival pathway and suppressing the TLR4/NF‐κB inflammatory pathway; on the other hand, it modulates gut microbiota to enhance the production of butyrate and other SCFAs, which subsequently enter the liver through the portal vein and synergistically inhibit the TLR4/NF‐κB pathway. Thus, ACCE alleviates AFB_1_‐induced liver injury through a multi‐target and multi‐pathway mechanism.

To validate this hypothesis, further in vitro studies were performed using HepG2 hepatocytes.

#### 2.6.1 ACCE Alleviates Hepatocyte Injury via the PI3K‐AKT Pathway

**FIGURE 7:**
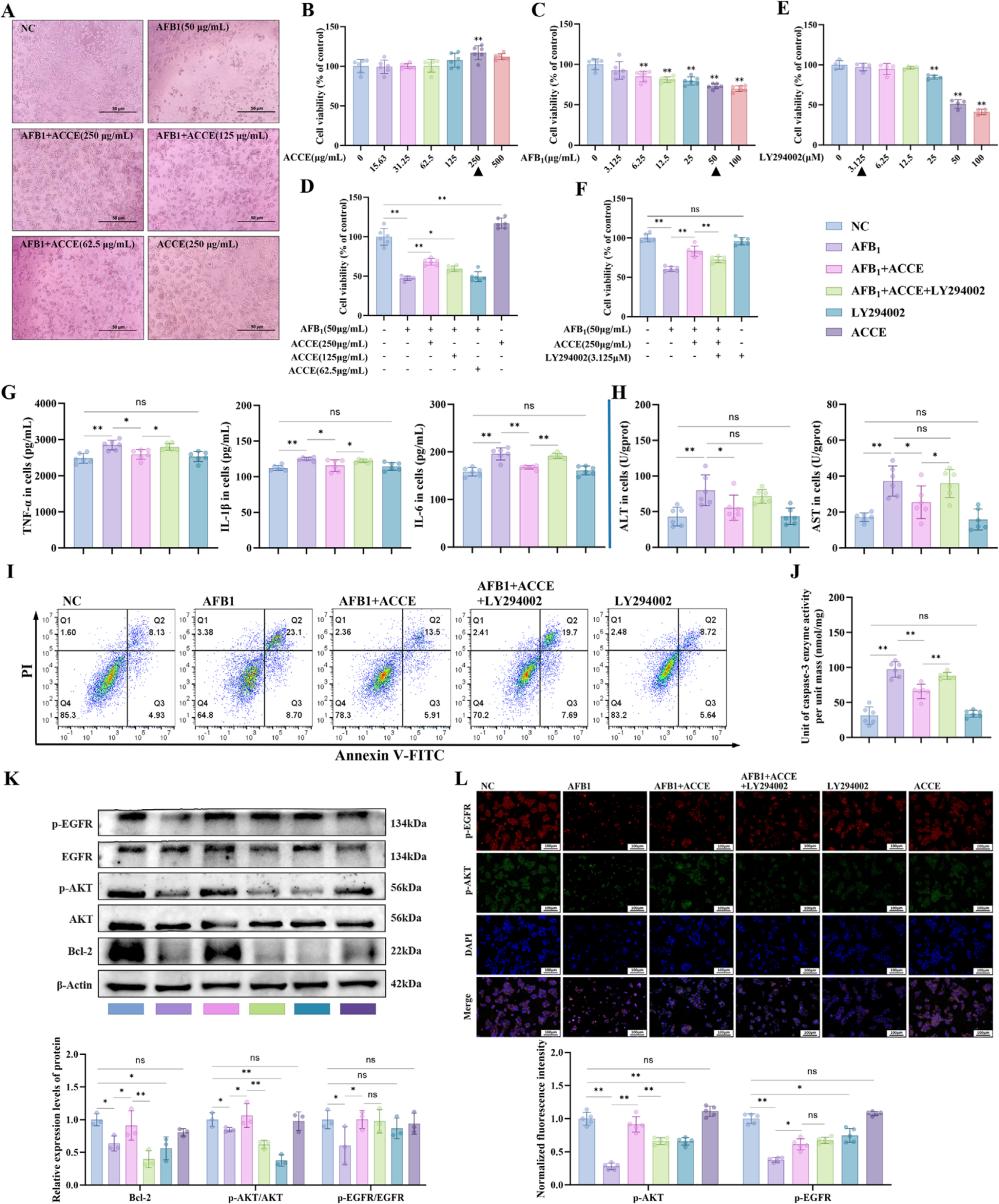
The effects of AFB_1_ exposure and the interventions of ACCE and LY294002 on HepG2. (A) Microscopic morphology of cells treated under different conditions (400×). (B) Safe concentrations of ACCE for cell treatment, *n* = 6. (C) IC50 of cells treated with AFB_1_, *n* = 6. (D) Cell viability after treatment with gradient concentrations of ACCE, *n* = 6. (E) Safe concentration of cells pretreated with LY294002, *n* = 4. (F) Cell viability after treatment with ACCE and LY294002 together, *n* = 6. (G) Levels of inflammatory factors in cells treated under different conditions, *n* = 6. (H) Liver function levels of cells treated with different conditions, *n* = 6. (I, J) Flow cytometry analysis of apoptosis and levels of apoptotic proteins in cells after different treatments, *n* = 6. (K) Western blotting detection of key proteins in the PI3K‐AKT pathway, *n* = 3. (L) Results of immunofluorescence detection of key proteins (400×), *n* = 5. Statistical analysis was performed by One‐Way ANOVA with Tukey post‐test (B‐H, J‐L) or Welch's ANOVA with Games‐Howell post‐test (G, L). ^*^ *p* < 0.05; ^**^ *p* < 0.01; ns, no significance.

To investigate the protective effect and underlying mechanism of ACCE against AFB_1_‐induced hepatocyte injury, in vitro experiments were conducted using HepG2 cells. CCK‐8 assay results showed that ACCE exhibited no cytotoxicity at concentrations ranging from 15.63 to 500 µg/mL. The half‐maximal inhibitory concentration (IC50) of AFB_1_ was 50 µg/mL, and ACCE dose‐dependently alleviated AFB_1_‐induced cellular injury (Figure 7A–D). Pretreatment with the broad‐spectrum PI3K inhibitor LY294002 (safe concentration: 0–12.5 µm) significantly attenuated the protective effect of ACCE. Compared with the AFB_1_‐only group, cells treated with ACCE showed significantly enhanced cell viability, decreased levels of inflammatory cytokines and liver function markers, reduced Caspase‐3 levels, and a decreased proportion of late‐apoptotic cells. However, these beneficial effects were partially reversed following pretreatment with LY294002 (Figure 7E–J). Furthermore, expression levels of key proteins within the PI3K‐AKT pathway (e.g., p‐AKT) were significantly elevated in the AFB_1_+ACCE group compared to the AFB_1_ group, but this elevation was abolished by LY294002 pretreatment (Figure 7K,L). Collectively, these findings indicate that ACCE mitigates AFB_1_‐induced apoptosis by activating the PI3K‐AKT signaling pathway in hepatocytes.

#### 2.6.2 Butyrate Alleviates Hepatocyte Inflammation via the TLR4/NF‐κB Pathway

**FIGURE 8:**
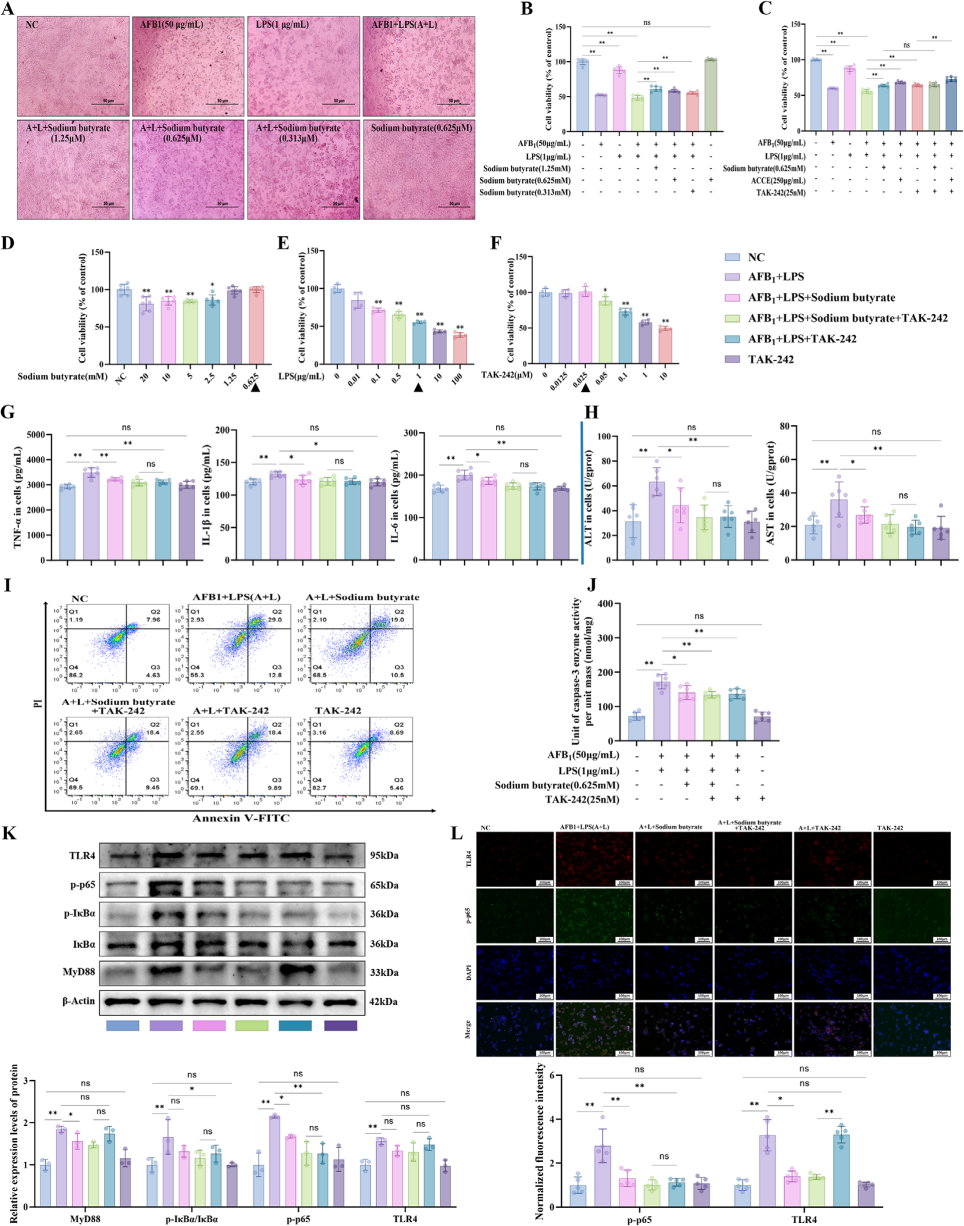
The effects of AFB_1_ and LPS exposure and the interventions of Butyrate and TAK‐242 on HepG2. (A) Microscopic morphology of cells treated under different conditions (400×). (B) Cell viability after treatment with gradient concentrations of Butyrate, *n* = 6. (C) Cell viability after treatment with Butyrate and TAK‐242 together, *n* = 6. (D) Safe concentrations of Butyrate for cell treatment, *n* = 6. (E) IC50 of cell treated with LPS, *n* = 4. (F) Safe concentration of cells pretreated with TAK‐242, *n* = 4. (G) Levels of inflammatory factors in cells treated under different conditions, *n* = 6. (H) Liver function levels of cells treated under different conditions, *n* = 6. I, (J) Flow cytometry analysis of apoptosis and levels of apoptotic proteins in cells after different treatments, *n* = 6. (K) Western blotting detection of key proteins in the TLR4/NF‐κB pathway, *n* = 3. (L) Results of immunofluorescence detection of key proteins (400×), *n* = 5. Statistical analysis was performed by One‐Way ANOVA with Tukey post‐test (B, D, F–H, K, L) or Welch's ANOVA with Games‐Howell post‐test (C, E, J, L). ^*^ *p* < 0.05; ^**^ *p* < 0.01; ns, no significance.

To further validate the anti‐inflammatory effects of butyrate and elucidate its underlying mechanisms in hepatocytes, an in vitro ‘AFB_1_+LPS’ combined stimulation model was established to mimic a “leaky gut” scenario, whereby hepatocytes are concurrently exposed to the exogenous toxin AFB_1_ and endotoxin LPS. The TLR4 inhibitor TAK‐242 was applied as a pretreatment agent. Preliminary tests determined that safe concentration ranges for butyrate and TAK‐242 in HepG2 cells were 0–1.25 mm and 0–0.025 µm, respectively (Figure 8D–F). Combined exposure to AFB_1_ and LPS significantly decreased cell viability compared with either agent alone, suggesting an additive hepatotoxic effect. Butyrate administration alleviated this combined injury in a dose‐dependent manner (Figure 8A,B). After TAK‐242 pretreatment, cell viability in the butyrate‐treated group showed no significant difference from the TAK‐242‐alone group, both of which were significantly higher than the AFB_1_+LPS group (Figure 8C). Additionally, groups treated with butyrate alone, TAK‐242 alone, or their combination exhibited significant improvements in inflammatory cytokine levels, liver function markers, Caspase‐3 levels, and apoptosis rates compared to the AFB_1_+LPS group, with no significant differences among these three treatment groups (Figure 8G,J). In addition, both butyrate and TAK‐242 treatments significantly suppressed the expression of key proteins within the TLR4/NF‐κB pathway (Figure 8K,L). These results collectively indicate that butyrate exerts anti‐inflammatory and hepatoprotective effects by inhibiting the TLR4/NF‐κB signaling pathway.

#### 2.6.3 Synergistic Effect of ACCE and Butyrate in Alleviating Hepatocyte Injury

**FIGURE 9:**
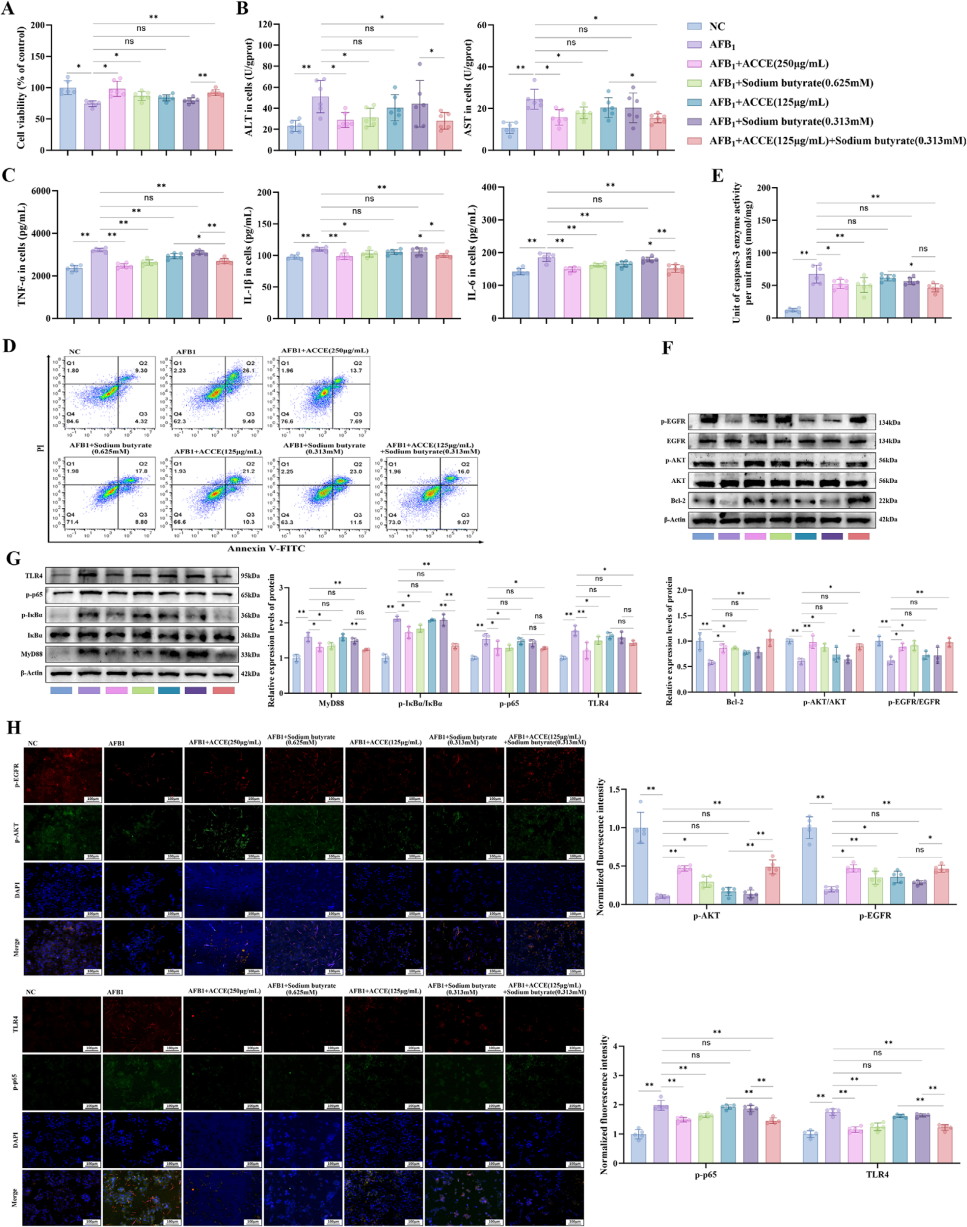
The effect of ACCE combined with butyrate treatment on HepG2 cells. (A) Effect of ACCE combined with butyrate treatment on cell viability, *n* = 6. (B) Liver function levels of cells treated under different conditions, *n* = 6. (C) Levels of inflammatory factors in cells treated with different conditions, *n* = 6. (D, E) Flow cytometry analysis of apoptosis and levels of apoptotic proteins in cells after different treatments, *n* = 6. (F, G) Western blotting was used to detect the expression levels of key proteins in the PI3K‐AKT pathway and TLR4/NF‐κB pathway, *n* = 3. (H) Results of immunofluorescence detection of key proteins (400×), *n* = 5. Statistical analysis was performed by One‐Way ANOVA with Tukey post‐test (B, C, F, G, H) or Welch's ANOVA with Games‐Howell post‐test (A, B, E, H). ^*^ *p* < 0.05; ^**^ *p* < 0.01; ns, no significance.

To investigate the synergistic effect of ACCE and butyrate in alleviating hepatocyte injury, a combination of half‐dose ACCE (125 µg/mL) and half‐dose butyrate (0.313 mm) was applied. The results demonstrated that, compared with the corresponding half‐dose single‐agent groups, the combination treatment significantly increased cell viability and produced more pronounced reductions in inflammatory cytokine levels, liver function markers, Caspase‐3 levels, and apoptotic cell proportions, with a protective effect comparable to that of the full‐dose ACCE group (Figure 9A–E). Regarding the regulation of key proteins in the PI3K‐AKT and TLR4/NF‐κB pathways, the combination group also exhibited stronger regulatory effects than the single‐agent half‐dose groups (Figure 9F–H). These findings indicate that ACCE and butyrate exert synergistic effects in suppressing liver inflammation and reducing hepatocyte apoptosis.

#### 2.6.4 Validation of Synergistic Effects among Functional Components of ACCE

Based on UPLC‐MS/MS analysis and network pharmacology predictions, three major components with high abundance in ACCE and strong binding affinity to core targets, caffeate, cryptotanshinone, and 6‐gingerol (all of which can be absorbed into the blood), were selected to evaluate their synergistic effects in alleviating liver injury [18, 19, 20].

Cell viability assays determined their safe concentration ranges to be 0–12.5 µm, 0–3.125 µm, and 0–12.5 µm, respectively. Concentrations that effectively alleviated AFB_1_‐induced hepatocyte injury (12.5 µm, 3.125 µm, and 12.5 µm) were defined as effective concentrations (ECs), whereas concentrations showing minimal or no protective effects when used individually (6.25 µm, 1.563 µm, and 3.125 µm) were defined as subeffective concentrations (SCs). A physical mixture of the three compounds at their SCs (subeffective concentration mixture, SCM) was prepared (Figure S2A,B).

The results showed that ACCE and the ECs of the three compounds significantly enhanced cell viability, whereas SC treatments alone showed no significant effect. In contrast, SCM treatment significantly increased cell viability (Figure S2C,D). Similarly, SCM treatment markedly reduced inflammatory cytokine levels, liver function markers, Caspase‐3 levels, and apoptotic cell proportions, and modulated the expression of key proteins in the PI3K‐AKT and TLR4/NF‐κB pathways, whereas SC treatments alone showed no significant effects (Figure S2E–K). These findings indicate that the principal active components of ACCE exert synergistic effects in alleviating hepatocyte injury.

#### 2.6.5 Cross‐Dimensional Synergy between Functional Components and Butyrate in Alleviating Hepatocyte Injury

To further explore the cross‐dimensional synergy between the main active components of ACCE and the microbial metabolite butyrate in alleviating liver injury, a new subeffective concentration mixture (NSCM) was constructed by combining SCM (SCs of caffeate, cryptotanshinone, and 6‐gingerol) with a subeffective concentration of butyrate.

The results demonstrated that ACCE, butyrate alone, and SCM treatment all significantly increased the viability of HepG2 cells after AFB_1_+LPS exposure, whereas the subeffective concentration of butyrate alone had no significant effect. Notably, NSCM treatment markedly enhanced cell viability (Figure S3A,B). Similarly, NSCM significantly reduced inflammatory cytokine levels, liver function markers, Caspase‐3 levels, and apoptotic cell proportions, and effectively regulated the expression of key proteins in the PI3K‐AKT and TLR4/NF‐κB pathways, with stronger effects than those observed with SCM or subeffective butyrate alone (Figure S3C–I). These results suggest that the hepatoprotective effects of ACCE arise from both intra‐component synergy among its active constituents and cross‐dimensional synergy between these components and the microbial metabolite butyrate.

### 2.7 Butyrate or Cryptotanshinone Can Alleviate AFB_1_‐Induced Liver Injury in Mice

Butyrate and Cryptotanshinone, which have a good protective effect on hepatocytes in cell assays, will be intervened in AFB_1_ exposed mice to further explore whether they are equally effective in vivo. The results showed that butyrate or Cryptotanshinone could reverse the up‐regulation of AFB_1_ in liver index, serum liver function indicators (ALT, AST), and inflammatory factors (TNF‐α, IL‐1β, IL‐6) levels in liver tissue. This finding indicated that either butyrate or Cryptotanshinone was effective in alleviating AFB_1_‐induced liver injury (Figure S5). The above in vivo results proved that the hepatoprotective effect of ACCE was related to its main active component, and butyrate, as an important player in the gut‐liver axis, also played an important role in the hepatoprotective process.

## 3 Discussion

According to existing data, AFB_1_ widely contaminates grain and animal feed globally and accumulates in animals and humans through the food chain, with the liver as its major target [21]. This contamination inflicts heavy economic losses on the livestock sector and simultaneously endangers human health, including the potential induction of liver cancer [22]. Currently, it is recognized that AFB_1_ induces liver injury mainly through two pathways that often occur concurrently. On one hand, AFB_1_ is metabolized by cytochrome P450 enzymes into epoxide derivatives, leading to oxidative stress, DNA damage, inflammation, and apoptosis, ultimately causing hepatocellular injury. Additionally, the release of damage‐associated molecular patterns (DAMPs) from injured liver tissues amplifies the inflammatory response [23, 24]. On the other hand, AFB_1_ compromises intestinal homeostasis by disrupting the mucosal barrier and tight junction proteins, leading to elevated permeability and subsequent LPS translocation to the liver, thereby intensifying inflammatory damage [7, 9, 25]. Therefore, novel agents that confer dual protection on both the liver and intestine are urgently needed to effectively counteract AFB_1_‐induced hepatic injury [26]. Our findings demonstrate that ACCE significantly alleviated AFB_1_‐induced hepatic injury and intestinal barrier dysfunction, with its protective effect closely associated with the synergistic actions of its main bioactive components and regulation of gut microbiota.

Consistent with previous findings, AFB_1_ exposure disrupted hepatocyte architecture and caused elevated liver function markers and oxidative stress [27, 28]. In this study, ACCE significantly decreased serum ALT and AST levels, mitigated oxidative stress, and demonstrated a pronounced hepatoprotective effect. Additionally, the intestinal barrier serves as a critical defense against harmful substances, playing an essential role in maintaining gut homeostasis [29, 30]. Numerous studies have indicated that AFB_1_ exposure not only induces liver injury but also impairs intestinal barrier function, with these two aspects closely interconnected. Here, ACCE markedly improved ileal morphological integrity, reduced intestinal permeability, and concurrently decreased serum LPS and inflammatory cytokine levels. These findings suggest that ACCE alleviates liver injury by restoring intestinal barrier function and preventing the systemic entry of detrimental substances such as LPS.

Network pharmacology, an essential tool in TCM research, can, to some extent, systematically predict the multi‐component and multi‐target therapeutic mechanisms of TCM formulations [31, 32, 33]. By integrating UPLC‐MS/MS analysis with network pharmacology, the present study identified 24 principal active components in ACCE (e.g., caffeate, cryptotanshinone, and 6‐gingerol) and screened 100 potential therapeutic targets related to AFB_1_‐induced liver injury (such as EGFR, STAT3, and MAPK1). Computer predictions suggested that the active constituents may bind strongly to the identified targets. GO and KEGG pathway analyses indicated that regulation of gene expression, apoptotic processes, and the PI3K‐AKT and MAPK cascades are potentially involved in the hepatoprotective action of ACCE against AFB_1_‐induced injury. Consequently, it was hypothesized that the protective effect of ACCE might be closely linked to its anti‐apoptotic activity, a mechanism requiring further experimental validation.

Integrative multi‐omics analysis is a powerful tool for elucidating drug mechanisms [34, 35, 36]. In this study, RNA‐seq data revealed that the DEGs were predominantly enriched in the PI3K‐AKT and Toll‐like receptor pathways, closely aligning with network pharmacology predictions. Results from 16S rRNA gene sequencing indicated that AFB_1_ exposure perturbed gut microbial homeostasis, whereas ACCE treatment effectively reshaped the microbial community by promoting beneficial taxa, including *Lachnospiraceae_NK4A136_group*, *norank_f_Lachnospiraceae*, and *norank_o_Clostridia_UCG‐014* [37, 38, 39]. Meanwhile, targeted metabolomic analysis demonstrated that levels of SCFAs (acetate, propionate, and butyrate) returned to normal after ACCE administration. Notably, a positive correlation was observed between butyrate levels and the relative abundance of *Lachnospiraceae_NK4A136_group*, a known SCFA producer. It was thus hypothesized that ACCE exerts its hepatoprotective effect by enriching SCFA‐producing bacteria, especially butyrate‐producing species, thereby elevating intestinal butyrate levels.

The consistency between network pharmacology predictions and transcriptomic findings often serves as a critical basis for exploring pharmacological mechanisms [40, 41, 42]. Integrating these analyses, the PI3K‐AKT pathway (governing cell proliferation and survival) and the TLR4/NF‐κB pathway (associated with inflammation) were hypothesized as key mechanisms in the protective action of ACCE against AFB_1_‐induced hepatic injury. This hypothesis was preliminarily supported by alterations in the expression levels of critical pathway proteins. Additionally, multi‐omics data demonstrated that ACCE significantly enriched intestinal SCFA‐producing bacteria and elevated butyrate concentrations. However, whether gut microbiota‐derived butyrate participates in ACCE‐mediated hepatoprotection, and the exact mechanism by which it acts, remained unclear. Previous studies have reported multiple physiological effects of butyrate, including anti‐inflammatory activity, maintenance of intestinal barrier function, and hepatoprotection, with its anti‐inflammatory effects primarily attributed to inhibition of the TLR4/NF‐κB signaling cascade [43, 44, 45, 46]. Notably, in our study, butyrate levels exhibited a significant negative correlation with inflammatory cytokines, serum LPS, and liver function markers, while a positive correlation was observed with intestinal tight junction protein expression. Thus, it was hypothesized that butyrate might serve as a critical signaling molecule connecting gut microbiota to liver protection, exerting its beneficial action by targeting the TLR4/NF‐κB signaling cascade in hepatocytes.

Integrating these findings, the following mechanistic hypothesis was proposed for ACCE‐mediated alleviation of AFB_1_‐induced liver injury: on one hand, active components of ACCE directly act upon hepatocytes to activate the PI3K‐AKT survival signaling pathway and suppress the TLR4/NF‐κB inflammatory axis; on the other hand, ACCE modulates gut microbiota, enriching SCFAs (particularly butyrate), which subsequently enter the liver via the portal vein and synergistically suppress the TLR4/NF‐κB signaling. Thus, ACCE alleviates liver injury through a multi‐target and multi‐pathway mechanism. This hypothesis was further verified through subsequent cell experiments.

In vitro studies visually demonstrated hepatocyte damage induced by AFB_1_ and the protective effects of ACCE and butyrate at the cellular level [47]. Upon exposure to AFB_1_, HepG2 cells exhibited morphological shrinkage, reduced viability, increased inflammatory cytokine levels and liver injury markers, upregulated apoptotic protein expression, and elevated proportions of late‐apoptotic cells. Treatment with ACCE significantly reversed these alterations and inhibited apoptosis; however, pretreatment with the PI3K inhibitor LY294002 weakened ACCE's anti‐apoptotic effects, and the ACCE‐induced increase in PI3K‐AKT pathway proteins was significantly diminished. These results confirmed that ACCE protects hepatocytes from AFB_1_‐induced apoptosis primarily through PI3K‐AKT pathway activation. To further determine whether butyrate mimics ACCE's anti‐inflammatory effects, a “leaky gut” model was created using co‐exposure to AFB_1_ and LPS in HepG2 cells, resulting in more severe injury than AFB_1_ alone. Similar to ACCE, butyrate significantly alleviated this injury and inhibited apoptosis. However, pretreatment with the TLR4 inhibitor TAK‐242 abolished butyrate's anti‐inflammatory effect (the anti‐inflammatory effects of butyrate and TAK‐242 alone were not significantly different). Upon TLR4 blockade, neither the pro‐inflammatory effects of LPS nor the anti‐inflammatory effects of butyrate occurred through the TLR4/NF‐κB axis, as protein levels of this pathway were uniformly lower across all treatment groups relative to the AFB_1_+LPS combination, further corroborating the pathway's involvement. Therefore, these findings indicate that butyrate, as a key mediator produced through ACCE‐induced modulation of gut microbiota, exerts its hepatoprotective effects predominantly through suppression of the TLR4/NF‐κB axis.

As a TCM compound, ACCE is rich in multiple active constituents, and network pharmacology predictions indicated robust binding affinity between these constituents and multiple targets. Therefore, a key question arises: is the hepatoprotection of ACCE against AFB_1_‐induced hepatic injury driven primarily by a single component, or do multiple components act synergistically? To address this issue, caffeate, cryptotanshinone, and 6‐gingerol, which exhibited high abundance in UPLC‐MS/MS analysis and strong binding affinity to core targets, were selected for further validation. When used individually at SC, the three components had minimal effects on AFB_1_‐induced elevations in inflammatory cytokines, abnormal liver function markers, Caspase‐3 levels, and apoptotic cell proportions. However, treatment with SCM significantly enhanced these protective effects. Similarly, SCM treatment significantly regulated the expression of PI3K‐AKT and TLR4/NF‐κB pathway proteins. These results suggest that the hepatoprotection of ACCE against AFB_1_‐induced hepatic injury stems from direct, cooperative regulation of hepatocytes by its multiple active components acting on diverse targets. Moreover, given that butyrate is a key effector molecule mediating the indirect hepatoprotective effect of ACCE, does it exhibit synergy with the primary active components of ACCE? To answer this, a new subeffective concentration mixture (NSCM) was constructed by combining SCM with a subeffective concentration of butyrate. Results revealed that NSCM exerted superior effects compared to SCM or butyrate alone, significantly improving inflammatory cytokines, liver function markers, Caspase‐3 levels, and apoptotic cell proportions. These data confirm that the hepatoprotection of ACCE against AFB_1_‐induced hepatic injury relies not only on synergy among its active components but also on cross‐dimensional synergy between these components and butyrate.

However, this study has limitations in research direction. For example, the liver is a complex organ composed of various cell types, including parenchymal cells (hepatocytes) and non‐parenchymal cells, which encompass hepatic stellate cells, Kupffer cells (resident hepatic macrophages), liver sinusoidal endothelial cells, and cholangiocytes [48, 49, 50]. These non‐parenchymal cells are critically involved in AFB_1_‐induced inflammation, fibrosis, and liver injury. As integral components of the liver's innate immunity, Kupffer cells release pro‐inflammatory mediators (e.g., TNF‐α, IL‐1β, IL‐6), promoting liver inflammation triggered by AFB_1_. Hepatic stellate cells are central to the process of fibrosis. Our in vitro experiments focused solely on hepatocytes and did not assess the effects of ACCE on non‐parenchymal cells. For instance, it remains unclear whether ACCE can inhibit the activation of Kupffer cells, alleviate macrophage‐mediated inflammatory cascades, or modulate the barrier function of liver sinusoidal endothelial cells. Therefore, the anti‐inflammatory and hepatoprotective effects of ACCE may involve the regulation of multiple liver cell types, and future studies may need to utilize co‐culture systems (such as hepatocytes and Kupffer cells) to further analyze the cell type‐specific actions of ACCE.

In addition, AFB_1_ requires bioactivation by cytochrome P450 enzymes (CYP450), primarily CYP1A2 and CYP3A4, to produce the reactive epoxide AFBO, which are the ultimate hepatotoxic and carcinogenic metabolite [51]. Consequently, numerous natural products have been shown to mitigate the hepatotoxicity of AFB_1_ by downregulating the expression and activity of these CYP450 subtypes, thereby reducing the metabolic activation of AFB_1_ [51, 52]. Given the complexity of the AFB_1_ metabolic network and our observation that ACCE has provided strong protective effects in subacute environments, we propose that future studies should systematically evaluate whether ACCE offers additional protective effects through the downregulation of hepatic CYP1A2 and CYP3A4 expression and activity in chronic, low‐dose exposure models. This would offer a more holistic perspective on the multi‐layered protective mechanisms of ACCE.

Similar to ACCE, which may act on a variety of cells. Increasing evidence suggests that AFB_1_‐induced liver injury is also regulated by multiple pathways or targets (e.g., HIPPO, PXR, NRF2 and NLRP3 inflammasome) rather than a single linear event [7, 11, 12, 13]. Our network pharmacology and transcriptomics analysis indicates that the PI3K‐AKT axis is a potential core pathway involved in AFB_1_ hepatotoxicity, which is consistent with recent network toxicology studies [53, 54]. This observation prompted us to select the PI3K‐AKT pathway as the core mechanism of ACCE's anti‐apoptotic effect for further investigation. Of course, other previously reported pathways, such as NRF2 and NLRP3, may also contribute to the hepatoprotection of ACCE, though this lies outside the scope of the present mechanistic investigation.

Notably, the regulation of the PI3K‐AKT pathway by AFB_1_ appears to vary across studies. Although our data show that AFB_1_ alone reduces hepatic p‐AKT levels in mice, which is consistent with several previously reported findings, some investigations have instead observed activation of this signaling axis following AFB_1_ exposure [55, 56, 57, 58]. The discrepancies in these results may arise from various factors, such as tissue and cell type specificity, differences in AFB_1_ dosage, and exposure duration, which could lead to divergent regulatory effects of AFB_1_ on the PI3K‐AKT pathway. Nevertheless, our data consistently demonstrate that under conditions of AFB_1_ exposure, ACCE can activate this pathway, which remains a robust and functionally significant finding.

In addition, butyrate is not limited to inhibiting TLR4/NF‐κB‐mediated inflammation; numerous previous studies have reported that butyrate also acts as a regulator of the PI3K‐AKT pathway. Butyrate attenuates cirrhotic portal hypertension in rats by suppressing HDAC3 and upregulating the PI3K/AKT/eNOS cascade within liver sinusoidal endothelial cells, as previously demonstrated [59]. A study on a diabetic mouse model explicitly indicated that the gut microbiota metabolite sodium butyrate (NaB) can positively influence bile acid metabolic homeostasis by activating the GPR43‐PI3K‐AKT‐GSK3 signaling pathway [60]. These findings corroborate the modulatory effect of butyrate on the PI3K‐AKT pathway. We thus propose that ACCE‐regulated, gut‐derived butyrate exerts hepatoprotection through a dual mechanism: it suppresses the TLR4/NF‐κB pathway to alleviate inflammation, and may also modulate the PI3K‐AKT pathway to promote cell survival. However, the more specific relationship of these actions still requires further investigation in the future.

Of course, there are some limitations in the experimental design of this study, such as: (1) First, the network pharmacology predictions lack experimental validation regarding the binding affinity of ACCE's major active constituents to the core targets. Therefore, the computational results should only be regarded as a reference for mechanism exploration. (2) Second, the HepG2 cell model selected in this study exhibits lower expression and activity of CYP450 compared to normal human primary hepatocytes, which may not fully simulate the metabolic activation process of AFB_1_ in human hepatocytes. Importantly, our use of HepG2 cells aims to validate signaling pathways rather than to accurately reproduce the complete metabolism of AFB_1_, so caution should be exercised when extrapolating the absolute sensitivity of AFB_1_ toxicity to normal human hepatocytes. Future validations using models with stronger metabolic capabilities, such as HepaRG or primary hepatocytes, will enhance the translational relevance of our findings. (3) Furthermore, while our in vitro inhibitor experiments (LY294002 and TAK‐242) validated the involvement of PI3K‐AKT and TLR4/NF‐κB pathways in ACCE‐mediated protection against AFB_1_‐induced hepatocyte injury, additional in vivo blockade studies are warranted to establish their causal roles in the intact liver. Therefore, it is essential to validate our findings in future studies using inhibitors or conditional gene knockout mice exposed to AFB_1_. (4) Lastly, it is important to note that the AFB_1_ exposure regimen used in this study (0.75 mg/kg bw, intragastric administration for 14 days) represents a widely used subacute/subchronic liver injury model, which has been similarly applied in previous AFB_1_ toxicology studies [53]. In contrast, the daily exposure level of AFB_1_ through dietary intake for residents in different regions of China is approximately 0.0066 to 0.0448 µg/kg.bw [61]. Thus, the dosage used in this study was significantly higher than the daily exposure level in humans, which aids in inducing reproducible liver injury within a shorter timeframe and aligns with the commonly used framework for subacute/subchronic toxicity assessment. Nevertheless, we acknowledge that this model cannot fully replicate the true scenario of chronic low‐dose exposure in humans; thus, future studies should utilize lower and more environmentally relevant doses, as well as longer exposure regimens, to further validate the clinical translational potential of ACCE.

In terms of the prospects and challenges of clinical translation, this study found that ACCE had a hepatoprotective effect against AFB_1_‐induced liver injury, suggesting its potential future as a feed additive or functional food for mitigating aflatoxin toxicity. However, several key issues need to be addressed before clinical translation. (1) The effective dose of ACCE in mice is 10 g of raw medicine/kg bw/day. Using the standard body surface area conversion formula, this dose translates to approximately 0.81 g/kg bw/day for humans (assuming a 60 kg adult), which amounts to about 48.6 g of raw medicine per day [62]. For livestock such as poultry or pigs, which are more relevant to AFB_1_ exposure, the corresponding dose can be determined based on allometric scaling of metabolic body weight. However, these estimates are preliminary and require direct validation in the target species. (2) ACCE is a traditional Chinese medicine compound aqueous extract with a long history of human use, indicating good safety [63]. In this study, no deaths, behavioral abnormalities, or weight loss were observed in mice treated with ACCE alone (10 g/kg bw/day) for 14 days, suggesting a reasonable safety window at the effective dose. However, chronic toxicity, genotoxicity, and reproductive toxicity studies are currently lacking, and these should be performed before human or livestock trials. (3) ACCE is prepared through a simple boiling method, making it cost‐effective, scalable, and compatible with feed production processes [64, 65]. Its protective effect against animal liver injury induced by AFB_1_, a major contaminant in feed, makes it a potential natural feed additive. Nevertheless, the stability of ACCE during feed storage and processing, as well as its impact on feed palatability and animal growth performance, still needs to be evaluated. In summary, while ACCE shows promise as a candidate for alleviating AFB_1_‐induced liver injury, extensive preclinical and translational research is still required before its application as a feed additive or functional food.

## 4 Conclusion

**FIGURE 10:**
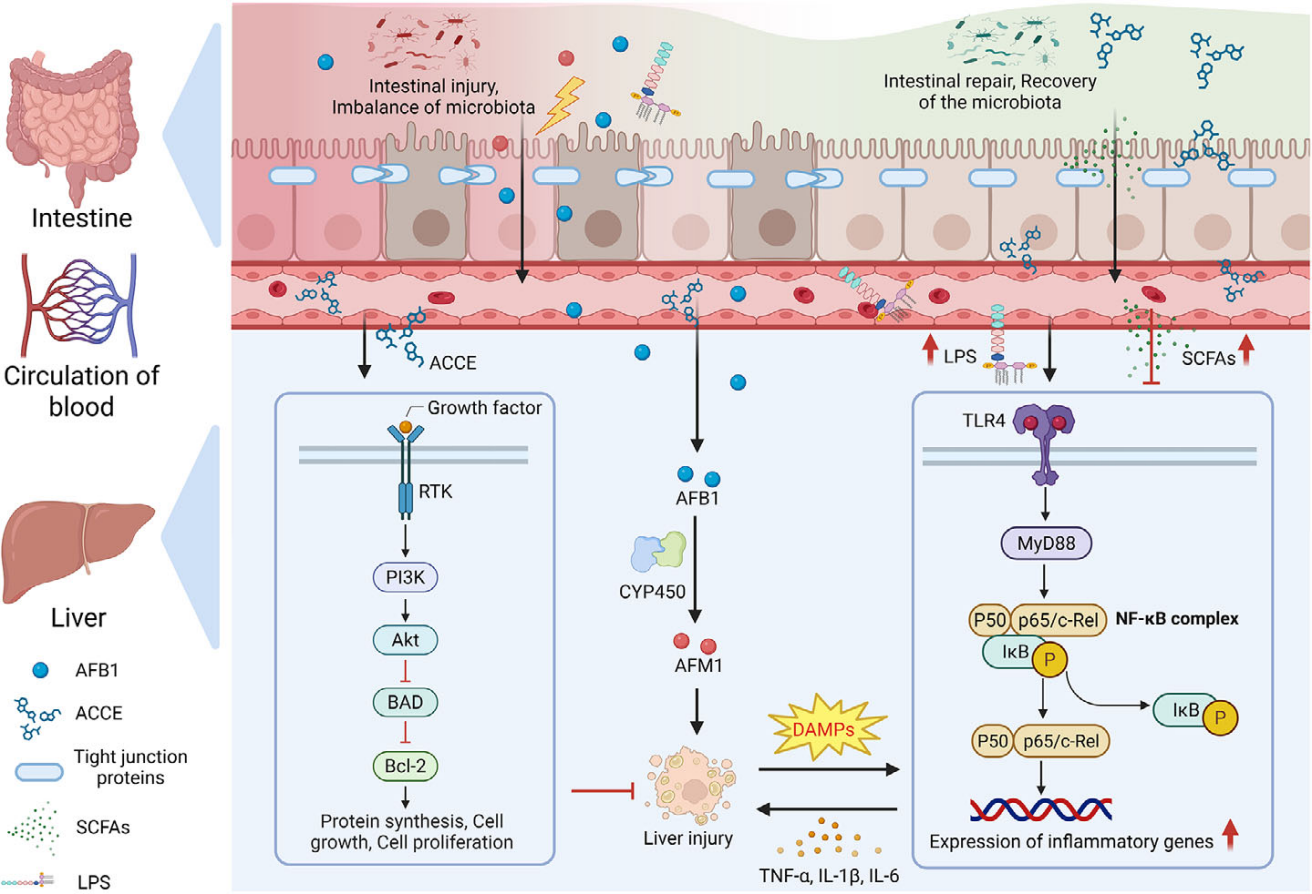
Mechanism of action of ACCE in alleviating liver injury. Created in BioRender. Hu, H. (2026) https://BioRender.com/o1ie23m.

In summary, ACCE demonstrates hepatoprotective potential against AFB_1_‐induced liver injury in mice, as revealed by this study. By integrating multi‐omics data with functional experiments, it reveals a novel mechanism by which ACCE alleviates AFB_1_‐induced liver injury. On one hand, active components of ACCE directly act upon hepatocytes, activating the PI3K‐AKT pathway and mediating anti‐apoptotic and anti‐inflammatory responses. Concurrently, ACCE reshapes gut microbiota composition by enriching butyrate‐producing bacteria, thereby increasing circulating butyrate levels. This butyrate may serve as a pivotal signaling molecule in gut‐liver crosstalk, reaching the liver via systemic circulation and contributing to anti‐inflammatory activity via TLR4/NF‐κB pathway inhibition. Together, these direct (components‐PI3K‐AKT) and indirect (microbiota‐butyrate‐TLR4/NF‐κB) pathways synergistically establish a multi‐target, multi‐dimensional protective network by which ACCE counteracts AFB_1_‐induced liver injury (Figure 10). This study offers experimental support for the alleviation of AFB_1_‐induced liver injury by ACCE, suggesting its potential value for further research. However, it should be emphasized that the long‐term safety, pharmacokinetic characteristics, and clinical translational potential of ACCE require validation in subsequent studies.

## 5 Materials and Methods

### 5.1 Materials and Reagents

The crude herbs of compound *Abrus cantoniensis* are provided by Hunan Songlingtang TCM Co., Ltd. (Hunan, China), and the quality control complies with the standards of the Chinese Pharmacopoeia. The specific batch numbers and origins are as follows: *Abrus cantoniensis* (Lot No. 241101, Hunan, China), *Artemisia capillaris* (Lot No. 240801, Shaanxi, China), *Salvia miltiorrhiza* (Lot No. 241002, Hebei, China), *Cynanchum otophyllum* (Lot No. 240802, Anhui, China), *Poriacocos* (Lot No. 240901, Hunan, China), and *Glycyrrhiza uralensis* (Lot No. 240902, Inner Mongolia, China). Silymarin (Cat# S873807), Butyrate (Cat# B766935), Cryptotanshinone (Cat# C796209), and 6‐Gingerol (Cat# C796096) were obtained from Shanghai Macklin Biochemical Technology Co., Ltd. (Shanghai, China). Lipopolysaccharide (LPS, Cat# S1732) and Caffeate (Cat# SM1022) were provided by Jiangsu Biyuntian Biotechnology Co., Ltd. (Jiangsu, China). LY294002 (Cat# HY‐10108) and TAK‐242 (Cat# HY‐11109) were supplied by Shanghai MCE (Shanghai, China). Aflatoxin B_1_ (AFB_1_, ≥99.8%) was purchased from Chengdu Lingli Biotechnology Co., Ltd. (Chengdu, China). Biochemical assay kits for alanine aminotransferase (ALT, Cat# C009‐2‐1), aspartate aminotransferase (AST, Cat# C010‐2‐1), alkaline phosphatase (ALP, Cat# AKFA018M), lactate dehydrogenase (LDH, Cat# AKCO003M), malondialdehyde (MDA, Cat# A003‐1), glutathione peroxidase (GSH‐Px, Cat# A005‐1), and superoxide dismutase (SOD, Cat# A001‐3) were provided by Nanjing Jiancheng Bioengineering Institute (Nanjing, China) and Beijing Boxbio Science & Technology Co., Ltd. (Beijing, China). Jiangsu Meimian Industrial Co., Ltd. (Jiangsu, China) supplied the ELISA kits for tumor necrosis factor‐α (TNF‐α, Cat# MM‐0122H1), interleukin‐1β (IL‐1β, Cat# MM‐0181H1), and interleukin‐6 (IL‐6, Cat# MM‐0049H1). Antibodies were obtained from Wuhan Servicebio Technology Co., Ltd. and Wuhan Proteintech Biotechnology Co., Ltd. (Wuhan, China). Majorbio Bio‐Pharm Technology Co., Ltd. (Shanghai, China) performed the transcriptomic, microbiomic, and targeted metabolomic analyses. The UPLC‐MS/MS analysis of ACCE was provided by Wuhan Maiwei Metabolomics Biotechnology Co., Ltd. (Wuhan, China).

### 5.2 Preparation of ACCE Extract

The six medicinal herbs comprising compound *Abrus cantoniensis* were crushed and thoroughly mixed. Ten times the weight (w/w) of purified water was added, and the mixture was soaked for 30 min, boiled, then simmered gently for 1 h. The decoction was filtered. The residue was re‐extracted with eight times the weight of purified water by gentle simmering for 1 h, followed by filtration. The two filtrates were pooled, centrifuged, and evaporated under reduced pressure to a concentration of 1 g crude drug/mL, yielding the compound ACCE. The concentration was adjusted according to experimental dosage requirements, after which the extract was aliquoted and kept at −20°C, and freshly prepared every 7 days. Any process that might produce microbial fermentation has been minimized. A portion of ACCE was lyophilized into powder (extraction rate of approximately 14.44%) for subsequent cell experiments.

### 5.3 UPLC‐MS/MS Analysis of ACCE

ACCE was analyzed using UPLC‐MS/MS. After extraction of samples with 70% methanol (containing an internal standard) and filtration of the supernatant, the extracts were subjected to Agilent SB‐C18 column chromatography with a gradient of water/acetonitrile (both with 0.1% formic acid), followed by MS/MS detection in MRM mode on an ESI‐QTRAP system. Metabolites were identified based on an in‐house database and quantified by integrating peak areas.

### 5.4 Network Pharmacology and Molecular Docking Analysis

Based on UPLC‐MS/MS results, combined with the TCMSP database and previous literature, the potential active components of ACCE were identified. Putative targets for each active component were predicted using the SwissTargetPrediction database. Subsequently, disease targets associated with AFB_1_‐induced liver disease (AILD) were retrieved from GeneCards and OMIM using search terms including “AFB_1_”, “liver disease”, and “liver injury”. Overlaps between compound‐related and disease‐related targets were then identified, and a “component‐target” interaction network was constructed with Cytoscape 3.10.2.

The overlapping targets were submitted to the STRING database to build a protein‐protein interaction (PPI) network, which was subsequently displayed using Cytoscape 3.10.2. CytoNCA plugin was applied to compute network topology parameters and screen hub targets. These candidates were then uploaded to DAVID for Gene Ontology (GO) and Kyoto Encyclopedia of Genes and Genomes (KEGG) pathway enrichment analyses. Results were ranked by p‐value and charted via the Weishengxin Online Platform (https://www.bioinformatics.com.cn/). A “drug‐component‐target‐pathway‐disease” network was constructed by integrating key targets and KEGG pathways.

Prior to molecular docking, ligands and target proteins were preprocessed (e.g., removal of water and addition of hydrogens). AutoDock Vina was then used to perform docking simulations, and binding energies were visualized as heatmaps. The interaction modes at the optimal binding sites were displayed using PyMOL.

### 5.5 Animals and Experimental Design

Male KM mice (4 weeks old, SPF grade, 20 ± 2 g) were obtained from Changsha Tianqin Biotechnology Co., Ltd. (Changsha, China; License No.: SCXK (Xiang) 2019‐0014). The experimental protocol was reviewed and approved by the Animal Ethics Committee of Guangxi University (Approval No.: Gxu‐2025‐231) and followed the National Research Council's Guide for the Care and Use of Laboratory Animals. All mice were allowed one week to acclimatize to the standard laboratory environment before the start of the experiment.

The mice were randomly allocated to seven groups (*n* = 11) and treated daily as follows: the normal control (NC) and AFB_1_ model groups (AFB_1_) received purified water by gavage; the vehicle control group (DMSO) received purified water by gavage; the low‐dose and high‐dose ACCE groups (AFB_1_+ACCE‐L, AFB_1_+ACCE‐H) received ACCE by gavage at doses of 5 g crude drug/kg body weight (bw) and 10 g crude drug/kg bw, respectively; the positive control group (Silymarin) received silymarin at 100 mg/kg bw by gavage (It has been shown to have a protective effect against AFB_1_‐induced liver injury) [66, 67]; and the ACCE‐alone group (ACCE) received ACCE at 10 g crude drug/kg bw by gavage. All groups received a gavage volume of 0.1 mL/10 g bw. Two hours after administration, except for mice in the NC and ACCE groups (gavaged with an equivalent volume of purified water) and the DMSO group (gavaged with an equivalent volume of 2% (v/v) DMSO), all other groups were given AFB_1_ (0.75 mg/kg bw) by gavage, and the intervention lasted for 14 days to establish an AFB_1_‐induced, repeatable subacute/subchronic liver injury model (Figure 1A) [7, 68]. This dose and duration are widely used in toxicological studies.

At the end of the treatment period, the mice were fasted overnight (12 h, water freely available) and weighed. Subsequently, the mice were anesthetized with pentobarbital sodium, and euthanasia was performed by cervical dislocation after blood collection. Liver, ileum, feces, and intestinal contents were promptly collected, snap‐frozen in liquid nitrogen, and stored at −80°C for subsequent analysis. Samples from ≥ 3 mice per group were randomly selected for detection and analysis based on data analysis requirements and objective experimental conditions (e.g., resource limitations).

In the in vivo validation experiment, mice were randomized into four groups (*n* = 6) and treated daily as follows: the normal control group (NC) and the AFB_1_ model group (AFB_1_) received pure water via gavage; the butyrate group (AFB_1_+Butyrate) and cryptotanshinone group (AFB_1_+Cryptotanshinone) were gavaged with 80 mg/kg and 25 mg/kg of the respective agents, all at 0.1 mL/10 g bw. Two hours later, except for the NC group, which was administered the same volume of pure water, the other groups were given 0.75 mg/kg bw AFB_1_ via gavage, with the intervention lasting for 14 days. After the treatment, samples were collected, preserved, and analyzed according to the procedures described above.

### 5.6 Histopathological Analysis

Liver and ileum tissues were fixed in 4% paraformaldehyde (24 h, room temperature), then dehydrated, paraffin‐embedded, and sectioned (3–4 µm). Sections were stained with hematoxylin and eosin (H&E), and images of target areas were captured using an Eclipse Ci‐L microscope (Nikon, Japan). Villus length and crypt depth in the ileum were measured with Image‐Pro Plus 6.0 software.

### 5.7 Biochemical Assays and Enzyme‐Linked Immunosorbent Assay (ELISA)

Biochemical assays were performed to measure serum liver function biomarkers (ALT, AST, ALP, LDH) and hepatic oxidative stress biomarkers (MDA, GSH‐Px, SOD) in tissue homogenates, following the respective manufacturers’ instructions. Inflammatory cytokines (TNF‐α, IL‐1β, IL‐6) in both serum and liver homogenates were quantified by ELISA.

### 5.8 RNA Sequencing (RNA‐seq) Analysis

Total RNA was isolated from mouse liver tissues using TRIzol reagent (Invitrogen, USA) according to the manufacturer's protocol. The concentration and purity were assessed on a NanoDrop 2000 (Thermo Fisher, USA), and RNA integrity was evaluated by agarose gel electrophoresis, with RQN values determined on an Agilent 5300. Subsequently, mRNA was enriched with Oligo (dT) magnetic beads, then fragmented, reverse‐transcribed into cDNA, ligated to adapters, and PCR‐amplified to generate the library. The quality‐controlled library was sequenced on an Illumina NovaSeq platform (PE150). Raw data were filtered to remove adapter sequences and low‐quality reads using fastp (v0.20.0). Clean reads were aligned to the mouse reference genome (GRCm39) using HISAT2 (v2.1.0). Illumina RNA‐seq and subsequent bioinformatics analyses were performed by Majorbio Bio‐Pharm Technology Co., Ltd. (Shanghai, China). Differentially expressed genes (DEGs) were identified using thresholds of false discovery rate (FDR) < 0.05 and |log_2_ (fold change)| ≥ 1 for subsequent functional analyses.

### 5.9 16S rRNA Gene Sequencing

Total genomic DNA was extracted from colon contents using the E.Z.N.A. Soil DNA Kit (Omega Bio‐tek, Norcross, GA, USA). DNA quality, concentration, and purity were assessed by 1% agarose gel electrophoresis and a NanoDrop 2000 spectrophotometer (Thermo Scientific, USA). Using the extracted DNA as a template, the bacterial 16S rRNA V3‐V4 region was amplified by PCR (20 µL reaction volume) with universal primers 338F (5’‐ACTCCTACGGGAGGCAGCAG‐3’) and 806R (5’‐GGACTACHVGGGTWTCTAAT‐3’) [69]. Amplified products were separated by 2% agarose gel electrophoresis, purified using a DNA Gel Recovery Purification Kit (Yuhua, China), and quantified using a Qubit 4.0 fluorometer (Thermo Fisher Scientific, USA). Libraries were constructed using the NEXTFLEX Rapid DNA‐Seq Kit and sequenced on an Illumina NextSeq 2000 platform (Majorbio Bio‐Pharm Technology Co., Ltd., Shanghai, China). Raw sequencing data were submitted to the NCBI SRA database. Following quality control and sequence assembly, sequencing data were denoised with the DADA2 plugin within the Qiime2 pipeline to generate amplicon sequence variants (ASVs) [70]. Subsequent α‐diversity and β‐diversity analyses were performed.

### 5.10 Short‐Chain Fatty Acid Quantification

Samples (approximately 20 mg) were extracted using an extraction solution at low temperature. After centrifugation, the supernatant was retained, and metabolites were further extracted with n‐butanol. Standard solutions with various concentrations were prepared and analyzed by GC‐MS under identical conditions. The concentration of target compounds was calculated based on standard curves and converted to actual content in samples.

### 5.11 Western Blot Analysis

Extraction of total proteins from frozen liver tissues was accomplished with RIPA buffer, while nuclear proteins were obtained using a nuclear extraction kit. Quantification of protein samples was carried out via the BCA method. SDS‐PAGE separation and PVDF membrane (Bio‐Rad) transfer were followed by immunodetection with specific antibodies. Prior to antibody incubation, membranes were blocked, then exposed to primary antibodies and subsequently to matching secondary antibodies. Visualization of target proteins was achieved by standard chemiluminescence detection, and band intensities were quantified with ImageJ software. Detailed antibody information is as follows: Anti‐p‐EGFR (Tyr1110, Bioss, Cat# bs‐5316R, 1:2000), Anti‐EGFR (Servicebio, Cat# GB111504, 1:1000), Anti‐p‐AKT (Ser473, Proteintech, Cat# 66444‐1, 1:5000), Anti‐AKT (Servicebio, Cat# GB15689, 1:1000), Anti‐Bcl‐2 (Servicebio, Cat# GB154830, 1:1000), Anti‐TLR4 (Servicebio, Cat# GB15186, 1:500), Anti‐p‐IκB (Ser32, Servicebio, Cat# GB15212, 1:1000), Anti‐IκB (Servicebio, Cat# GB151509, 1:1000), Anti‐MyD88 (Servicebio, Cat# GB111554, 1:1000), Anti‐p‐NF‐κB p65 (Ser536, Proteintech, Cat# 80379‐2, 1:5000), Anti‐β‐Actin (Servicebio, Cat# GB15001, 1:3000), Anti‐β‐Tubulin (Servicebio, Cat# GB15140, 1:1000), HRP‐labeled goat anti‐mouse IgG (Servicebio, Cat# GB23301, 1:10000), HRP‐labeled goat anti‐rabbit IgG (Servicebio, Cat# GB23303, 1:10000).

### 5.12 Immunofluorescence Analysis

Paraffin‐embedded liver tissues (sectioned at 5 µm thickness) or HepG2 cells cultured on coverslips were first permeabilized with 0.2% Triton X‐100 and blocked with 5% bovine serum albumin (BSA), then incubated overnight at 4 °C with primary antibodies. Subsequent incubation with fluorescent secondary antibodies was carried out for 1 h at room temperature in the dark, and DAPI was used for nuclear counterstaining. Images were captured using a fluorescence microscope and analyzed quantitatively with Saiviewer software. The relevant antibody information is, primary antibody: Anti‐p‐EGFR (Tyr1110, Bioss, Cat# bs‐5316R, 1:400), Anti‐p‐AKT (Ser473, Proteintech, Cat# 66444‐1, 1:600), Anti‐p‐NF‐κB p65 (Ser536, Proteintech, Cat# 80379‐2, 1:600), Anti‐NF‐κB p65 (Proteintech, Cat# 80979‐1, 1:600), 1:500), Anti‐TLR4 (Proteintech, Cat# 66350–1, 1:500); Secondary antibody: Alexa Fluor 488‐labeled goat anti‐mouse IgG (Servicebio, Cat# GB25301, 1:400) and CY3‐labeled goat anti‐mouse IgG (Servicebio, Cat# GB21301, 1:400) 1:300), Alexa Fluor 488‐labeled goat anti‐rabbit IgG (Servicebio, Cat# GB25303, 1:400), and CY3‐labeled goat anti‐rabbit IgG (Servicebio, Cat# GB21303, 1:300).

### 5.13 Cell Culture

HepG2 cells, due to their clear genetic background, rapid growth, lack of differentiation requirements, and ease of handling, are commonly used as an in vitro model for AFB_1_ hepatotoxicity studies, facilitating labor‐intensive cellular experiments [71, 72, 73, 74, 75, 76, 77, 78]. Seeding of cells into 96‐well plates was carried out at 5 × 10^4^ cells/well, followed by culture in DMEM (10% FBS, 100 U/mL penicillin, 100 µg/mL streptomycin) at 37°C in a 5% CO_2_ incubator. AFB_1_, caffeate, cryptotanshinone, and 6‐gingerol were dissolved in DMSO, with the final solvent concentration kept below 0.2% (v/v) in all treatment groups. Cells were allowed to acclimate for 24 h before treatment and subsequently exposed to AFB_1_, LY294002, TAK‐242, ACCE, butyrate, caffeate, cryptotanshinone, or 6‐gingerol as specified. Cell viability, inflammatory cytokines, liver function markers, apoptosis, and protein expression were respectively determined by CCK‐8 assay, ELISA/biochemical kits, flow cytometry, and Western blotting following the intervention. All cell experiments were conducted with at least three independent biological replicates, each containing a minimum of three technical replicates. The average of the technical replicates was taken as one data point per biological replicate for subsequent statistical analysis.

### 5.14 Flow Cytometry Analysis

Evaluation of apoptosis was performed using the Annexin V‐FITC/PI kit (Servicebio, Cat# G1511). Following culture, HepG2 cells were collected, washed twice with pre‐cooled PBS, followed by adjusting the cell concentration to 1 × 10^6^ cells/mL using pre‐cooled 1× Binding Buffer. The cell suspension (100 µL) was incubated with 5 µL each of Annexin V‐FITC and PI for 10 min at room temperature in the dark. Stained cells were analyzed on a flow cytometer (Attune Nxt) within 1 h, acquiring 20,000 events per sample. For gating, debris were first excluded by FSC‐A/SSC‐A selection, and quadrants were then defined. Apoptotic cells were classified as Annexin V^+^/PI^−^ (early) or Annexin V^+^/PI^+^ (late), with data processed using FlowJo v10.

### 5.15 Statistical Analysis

Presentation of data as mean ± SD from ≥3 independent experiments was adopted. Verification of normality and homogeneity of variances was accomplished via Shapiro‐Wilk and Levene's tests, respectively. One‐way ANOVA with Tukey's post hoc correction was applied to datasets fulfilling both assumptions (*p* > 0.05), whereas Welch's ANOVA with Games‐Howell adjustment was employed for datasets with unequal variances. Importantly, both analytical strategies yielded concordant conclusions for all principal comparisons. SPSS 21.0 (SPSS Inc., Chicago, IL, USA) and GraphPad Prism 10 (GraphPad Software, San Diego, CA, USA) were used for statistical processing, with the threshold for significance fixed at *p* < 0.05.

## Author Contributions

H.H.: formal analysis, supervision, methodology, Writing – original draft, and funding acquisition. M.C., S.Z., and H.L.: formal analysis, validation, methodology, and Writing – review & editing. X.W., W.Y., H.Y and L.Y.: validation, Writing – review & editing. L.J.: translation and polishing of the manuscript. J.C., L.Z., and Y.G.: Writing – review & editing and supervision. H.S.: project administration, supervision, funding acquisition, and Writing – review & editing.

## Conflicts of Interest

The authors declare no conflicts of interest.

## Supporting information




**Supporting File**: advs77889‐sup‐0001‐SuppMat.docx.

## Data Availability

The data that support the findings of this study are available from the corresponding author upon reasonable request.
