# Supplementary material for: Compound Abrus cantoniensis Extract Alleviates AFB1‐Induced Liver Injury by Regulating PI3K‐AKT Pathway and Gut Microbiota/Butyrate Metabolism
Source: Adv Sci (Weinh). 2026 Sep 27:e77889. Online ahead of print. doi: 10.1002/advs.77889 (PMC13616414; doi:10.1002/advs.77889)
Supplement: Supplementary file 1 [file ADVS-9999-e77889-s001.docx]

****Supporting Information****

**Compound *Abrus cantoniensis* Extract alleviates AFB₁-induced liver injury by regulating PI3K-AKT pathway and gut microbiota/butyrate metabolism**

*Hongjie Hu, Ming cheng, Shiqi Zheng, Huixin Liu, Xiaofang Wei, Wenwen Yang, Hongchun Yang, Liuyang Yuan, Li Jiang, Jie Chen, Linglong Zhao, Yanchao Guo, and Hongbin Si**

Supplementary Figures

**Figure S1.** Composition of ACCE and results of UPLC-MS/MS analysis. A) The herbs that make up ACCE. B) UPLC-MS/MS analysis. C, D) Total ion current map (TIC) of mixed quality control QC samples.

**Figure S2.** The effects of AFB1 exposure and the functional component treatment of ACCE on HepG2 cells. A) Safe concentrations of Caffeate, Cryptotanshinone, and 6-Gingerol in cells, n = 4. B) Cell viability after treatment of cells with different concentrations of the three components, n = 6. C) Microscopic morphology of cells treated with different conditions (400×). D) Cell activity after treatment of cells with SC and SCM of 3 components, n = 6. E) Levels of inflammatory factors in cells treated with different conditions, n = 6. F) Liver function levels of cells treated with different conditions, n = 6. G, H) Flow cytometry analysis of apoptosis and levels of apoptotic proteins in cells after different treatments, n = 6. I, J) Western blotting was used to detect the expression levels of key proteins in PI3K-AKT pathway and TLR4/NF-κB pathway, n = 3. K) Results of immunofluorescence detection of key proteins (400×), n = 5. Statistical analysis was performed by One-Way ANOVA with Tukey post-test (A, B, E, F, H-K) or Welch’s ANOVA with Games-Howell post-test (D, K). ^*^ *P* ＜ 0.05; ^**^ *P* ＜ 0.01; ns, no significance.

**Figure S3.** The effects of functional components combined with butyrate treatment on HepG2 cells. A) Microscopic morphology of cells treated with different conditions (400×). B) Cell viability after SCM combined with butyrate treatment of cells, n = 6. C) Levels of inflammatory factors in cells treated with different conditions, n = 6. D) Liver function levels of cells treated with different conditions, n = 6. E, F) Flow cytometry analysis of apoptosis and levels of apoptotic proteins in cells after different treatments, n = 6. G, H) Western blotting was used to detect the expression levels of key proteins in PI3K-AKT pathway and TLR4/NF-κB pathway, n = 3. I) Results of immunofluorescence detection of key proteins (400×), n = 5. Statistical analysis was performed by One-Way ANOVA with Tukey post-test (C, D, G-I) or Welch’s ANOVA with Games-Howell post-test (B, F). ^*^ *P* ＜ 0.05; ^**^ *P* ＜ 0.01; ns, no significance.

**Figure S4.** Results of butyrate assay in peripheral blood of mice. A) Butyrate concentration in the serum of mice, n = 6. Statistical analysis was performed by One-Way ANOVA with Tukey post-test (A). ^*^ *P* ＜ 0.05, ^**^ *P* ＜ 0.01, versus NC; ^#^ *P* ＜ 0.05, ^##^ *P* ＜ 0.01, versus AFB_1_.

**Figure S5.** Effects of AFB_1_ exposure and butyrate or cryptotanshinone intervention on liver index, function, and inflammation in mice. A) Schematic representation of AFB_1_, butyrate, and cryptotanshinone treatment in animal experiments. B) Liver organ index, n = 6. C) Butyrate concentration in the serum of mice, n = 6. D) Detection results of liver function index, n = 6. E) Related indicators of liver inflammation, n = 6. F) The level of LPS in the serum, n = 6. Statistical analysis was performed by One-Way ANOVA with Tukey post-test (B, D-F) or Welch’s ANOVA with Games-Howell post-test (C). ^*^ *P* ＜ 0.05, ^**^ *P* ＜ 0.01, versus NC; ^#^ *P* ＜ 0.05, ^##^ *P* ＜ 0.01, versus AFB_1_.

Supplementary Table

**Table S1.** The 24 major components in ACCE.

| No. | Molecular weight (Da) | Formula | Ionization model | CAS | Area | Compounds | RT (min) | Level |
| --- | --- | --- | --- | --- | --- | --- | --- | --- |
| 1 | 718.1534 | C36H30O16 | [M-H]- | 115939-25-8 | 1.09E+08 | Salvianolic acid B* | 4.5 | 1 |
| 2 | 354.0951 | C16H18O9 | [M-H]- | 906-33-2 | 9.73E+07 | Neochlorogenic acid* | 2.58 | 1 |
| 3 | 354.0951 | C16H18O9 | [M-H]- | 327-97-9 | 8.69E+07 | Chlorogenate* | 2.74 | 1 |
| 4 | 564.1479 | C26H28O14 | [M+H]+ | 52012-29-0 | 7.61E+07 | Isoschaftoside | 3.52 | 1 |
| 5 | 278.1518 | C16H22O4 | [M+H]+ | 17851-53-5 | 7.46E+07 | Butyl isobutyl phthalate* | 9.58 | 1 |
| 6 | 594.1585 | C27H30O15 | [M+H]+ | 195450-50-1 | 5.17E+07 | Chiirirhamnin | 3.12 | 1 |
| 7 | 318.1103 | C17H18O6 | [M+H]+ | 697300-41-7 | 4.81E+07 | Paeoniflorgenin | 3.32 | 1 |
| 8 | 198.0528 | C9H10O5 | [M-H]- | 76822-21-4 | 4.60E+07 | (R)-3-(3,4-Dihydroxyphenyl)lactate | 2.16 | 1 |
| 9 | 243.0855 | C9H13N3O5 | [M+H]+ | 147-94-4 | 4.33E+07 | Cytarabine | 1.34 | 1 |
| 10 | 464.0955 | C21H20O12 | [M+H]+ | 482-36-0 | 4.33E+07 | Hyperin* | 3.91 | 1 |
| 11 | 296.1412 | C19H20O3 | [M+H]+ | 35825-57-1 | 4.06E+07 | Cryptotanshinone | 9.24 | 1 |
| 12 | 180.0423 | C9H8O4 | [M-H]- | 331-39-5 | 3.93E+07 | Caffeate | 3.38 | 1 |
| 13 | 138.0317 | C7H6O3 | [M-H]- | 99-96-7 | 3.91E+07 | 4-Hydroxybenzoate | 3.05 | 1 |
| 14 | 464.0955 | C21H20O12 | [M+H]+ | 482-35-9 | 3.68E+07 | Isoquercitrin* | 3.95 | 1 |
| 15 | 354.0951 | C16H18O9 | [M-H]- | 1241-87-8 | 3.47E+07 | 1-Caffeoylquinic acid | 2.21 | 1 |
| 16 | 136.0524 | C8H8O2 | [M-H]- | 99-93-4 | 3.17E+07 | 4'-Hydroxyacetophenone* | 4 | 1 |
| 17 | 516.1268 | C25H24O12 | [M-H]- | 14534-61-3 | 3.10E+07 | Isochlorogenic acid B | 4.16 | 1 |
| 18 | 516.1268 | C25H24O12 | [M-H]- | 57378-72-0 | 3.06E+07 | Isochlorogenic acid C* | 4.04 | 1 |
| 19 | 251.1018 | C10H13N5O3 | [M+H]+ | 73-03-0 | 3.04E+07 | Cordycepin* | 2.06 | 1 |
| 20 | 154.0266 | C7H6O4 | [M-H]- | 99-50-3 | 2.84E+07 | 3,4-Dihydroxybenzoate* | 2.55 | 1 |
| 21 | 294.1831 | C17H26O4 | [M+H]+ | 23513-14-6 | 2.76E+07 | [6]-Gingerol | 7.02 | 1 |
| 22 | 646.3717 | C36H54O10 | [M+H]+ | 34096-83-8 | 2.61E+07 | Glycyrrhetic acid monoglucuronide | 6.42 | 1 |
| 23 | 312.2301 | C18H32O4 | [M-H]- | 34444-18-3 | 2.45E+07 | Hydroperoxylinoleic acid* | 7.31 | 1 |
| 24 | 494.1213 | C26H22O10 | [M-H]- | 96574-01-5 | 2.42E+07 | Salvianolic acid A | 4.76 | 1 |
